# Characterization of a highly specific NQO1-activated near-infrared fluorescent probe and its application for in vivo tumor imaging

**DOI:** 10.1038/s41598-019-44111-8

**Published:** 2019-06-12

**Authors:** Surendra Reddy Punganuru, Hanumantha Rao Madala, Viswanath Arutla, Ruiwen Zhang, Kalkunte S. Srivenugopal

**Affiliations:** 1 Department of Pharmaceutical Sciences, School of Pharmacy, Texas Tech University Health Sciences Center, Amarillo, TX 79106 USA; 2 Department of Pharmacological and Pharmaceutical Sciences, College of Pharmacy, University of Houston, Houston, TX 77204 USA

**Keywords:** Cancer imaging, Diagnostic markers

## Abstract

The Near-infrared Fluorescence (NIRF) molecular imaging of cancer is known to be superior in sensitivity, deeper penetration, and low phototoxicity compared to other imaging modalities. In view of an increased need for efficient and targeted imaging agents, we synthesized a NAD(P)H quinone oxidoreductase 1 (NQO1)-activatable NIR fluorescent probe (NIR-ASM) by conjugating dicyanoisophorone (ASM) fluorophore with the NQO1 substrate quinone propionic acid (QPA). The probe remained non-fluorescent until activation by NQO1, whose expression is largely limited to malignant tissues. With a large Stokes shift (186 nm) and a prominent near-infrared emission (646 nm) in response to NQO1, NIR-ASM was capable of monitoring NQO1 activity *in vitro* and *in vivo* with high specificity and selectivity. We successfully employed the NIR-ASM to differentiate cancer cells from normal cells based on NQO1 activity using fluorescence microscopy and flow cytometry. Chemical and genetic approaches involving the use of ES936, a specific inhibitor of NQO1 and siRNA and gene transfection procedures unambiguously demonstrated NQO1 to be the sole target activating the NIR-ASM in cell cultures. NIR-ASM was successfully used to detect and image the endogenous NQO1 in three live tumor-bearing mouse models (A549 lung cancer, Lewis lung carcinoma, and MDMAMB 231 xenografts) with a high signal-to-low noise ratiometric NIR fluorescence response. When the NQO1-proficient A549 tumors and NQO1-deficient MDA-MB-231 tumors were developed in the same animal, only the A549 malignancies activated the NIR-ASM probe with a strong signal. Because of its high sensitivity, rapid activation, tumor selectivity, and nontoxic properties, the NIR-ASM appears to be a promising agent with clinical applications.

## Introduction

Biomedical imaging plays an important role in all phases of cancer management including screening, guidance for biopsy excisions, malignancy staging, prognosis and therapy planning, follow-up and in looking for early responses to cancer treatments and to identify patients who are not responding to therapy^1^. In addition to cancer, the imaging technology has wide applications in angiography, treatment of gastrointestinal and kidney diseases^2^. With solid tumors, the detection threshold of current imaging technology is approximately 10^9^ cells (1 g = 1 cm^3^) growing as a single mass. Fluorescence imaging appears to be superior in sensitivity and resolution compared to other imaging techniques for early detection of cancer biomarkers, but high background signal hinders the application^3,4^. One way to avoid such background noise is to develop activatable fluorescent probes, which remain non-fluorescent until being activated by tumor-specific molecular targets^5^. The enzyme-activated, particularly reductases enabled “turn-on” probes can generate rapid, highly sensitive, and selective signals associated with cancer cells, and this type of probes has seen growing interest^6–8^. One such cancer-specific target enzyme is the NAD(P)H quinone oxidoreductase 1 (NQO1) and accurate detection of NQO1 activity with high sensitivity and selectivity is useful for the early diagnosis of cancer^9^.

NQO1 is a two-electron reductase responsible for the detoxification of xenobiotics such as quinones and abnormally overexpressed in many tumors and intimately linked with multiple carcinogenic processes^10^. The increased oxidative stress inherent in human cancers has been assumed to underlie the higher expression of NQO1, which at the same time can help to cope with the elevated redox alteration^11^. Recent studies indicated that compared to healthy tissue, NQO1 expressed at levels of 5−200-fold above in tumors, such as the breast^12^, lung^13,14^, prostate^15^, stomach^16^, colon^17^, pancreas^18^, head, and neck cancer^19^. Consistent with this, NQO1 has been extensively explored to achieve highly selective and sensitive detection or visualization of tumor-associated events in different cancers. Most of the NQO1 activated fluorescent probes were developed based on its property of quinone bio-reduction^20^, where the fluorophore-conjugated with the quinone-based NQO1 substrates are held in a quenched non-fluorescent state by a trigger group. Intense fluorescence of these probes is unveiled upon removal of the quinone moiety by NQO1. McCarley and co-workers first identified the “trimethyl lock” containing quinone propionic acid (QPA) as an excellent NQO1-responsive trigger group and validated its specificity against NQO1^21^. Accordingly, several QPA-based fluorescent probes for detection and imaging of NQO1 in cancer cells. The fluorophores used in these studies include rhodamines^22,23^, napthalimides^24–26^, Acedan^27^, coumarin^28^, HO-BODIPY^29^, tetraphenylethan^30^, amino-acetyl-naphthalene^31^ and carbocyanine^32,33^. On the other hand, several affinity-based small molecule probes have been developed by linking the NQO1 inhibitor as the recognition group^34–36^. All these fluorescent probes have shown good selectivity even in the presence of other cellular reductants. However, their use for real-time detection of NQO1 in biological systems, a potentially effective approach for cancer diagnosis is limited because of their short and overlapping wavelengths of excitation and emission. To overcome these limitations recently we have developed an NQO1 activated fluorescent probe NQ-DCP with large Stokes shift for cancer cell imaging^37^. Although NQ-DCP displayed high sensitivity and selectivity against NQO1 it has several limitations for *in vivo* applications. These limitations including (1) The presence of ester bond, which is less stable at philological conditions; (2) signal to background ratio is low with auto-fluorescence; (3) Not applicable for *in-vivo* applications particularly with local and disseminated tumors because of low penetration of fluorescence light; (4) failed in *in vivo* imaging of lung cancer in orthotopic models.

**Figure 1:**
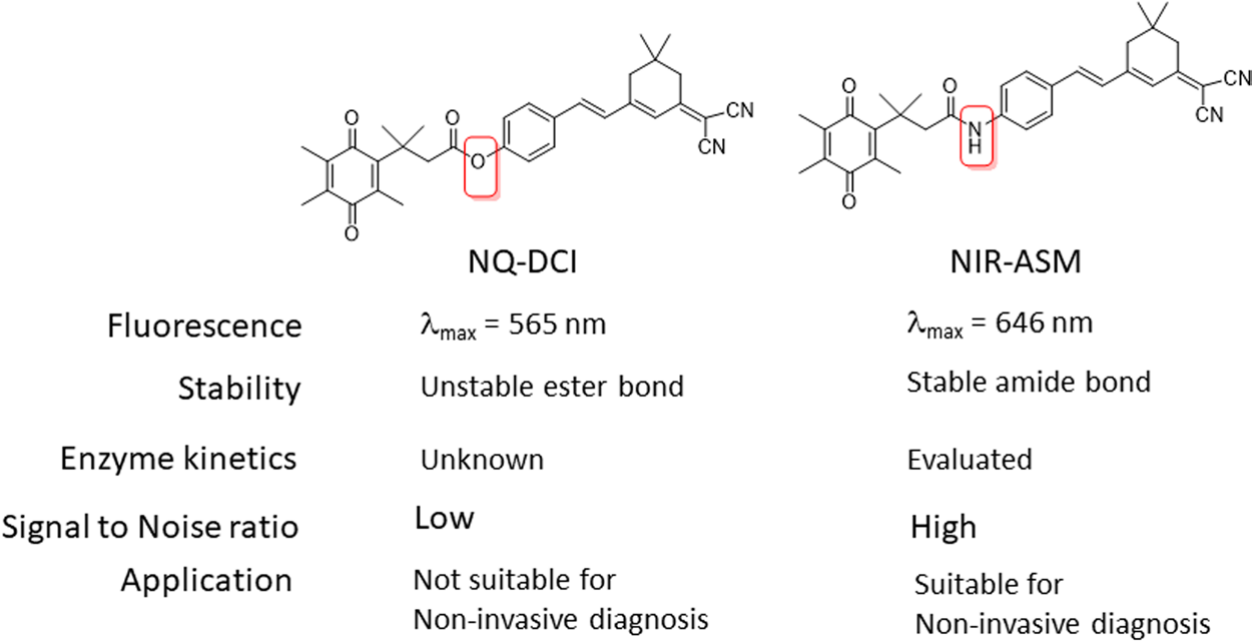
Structure and application differences between NQ-DCP and NIR-ASM.

Near-infrared fluorescent (NIR) probes have distinct advantages over traditional fluorescent probes and become increasingly popular tools in the field of bioimaging^38^. The properties including low absorption of the NIR region by biological molecules leads to dramatically reduced levels of autofluorescence and deeper penetration into body tissues^39^. Despite the great implication of NQO1 as a biomarker for early diagnosis of cancer, none of the reported fluorescent probes have been evaluated for the non-invasive diagnosis of cancer in orthoptic cancer xenograft models. Taking these points into consideration and to overcome the limitations of NQ-DCP, we developed a physiologically stable new NQO1 activatable ‘turn-on’ near-infrared fluorescent probe (NIR-ASM) for monitoring endogenous NQO1 activity and noninvasive cancer diagnosis (Fig. 1).

## Results and Discussion

### Design, preparation, and characterization of the NIR-ASM fluorescent probe

**Figure 2:**
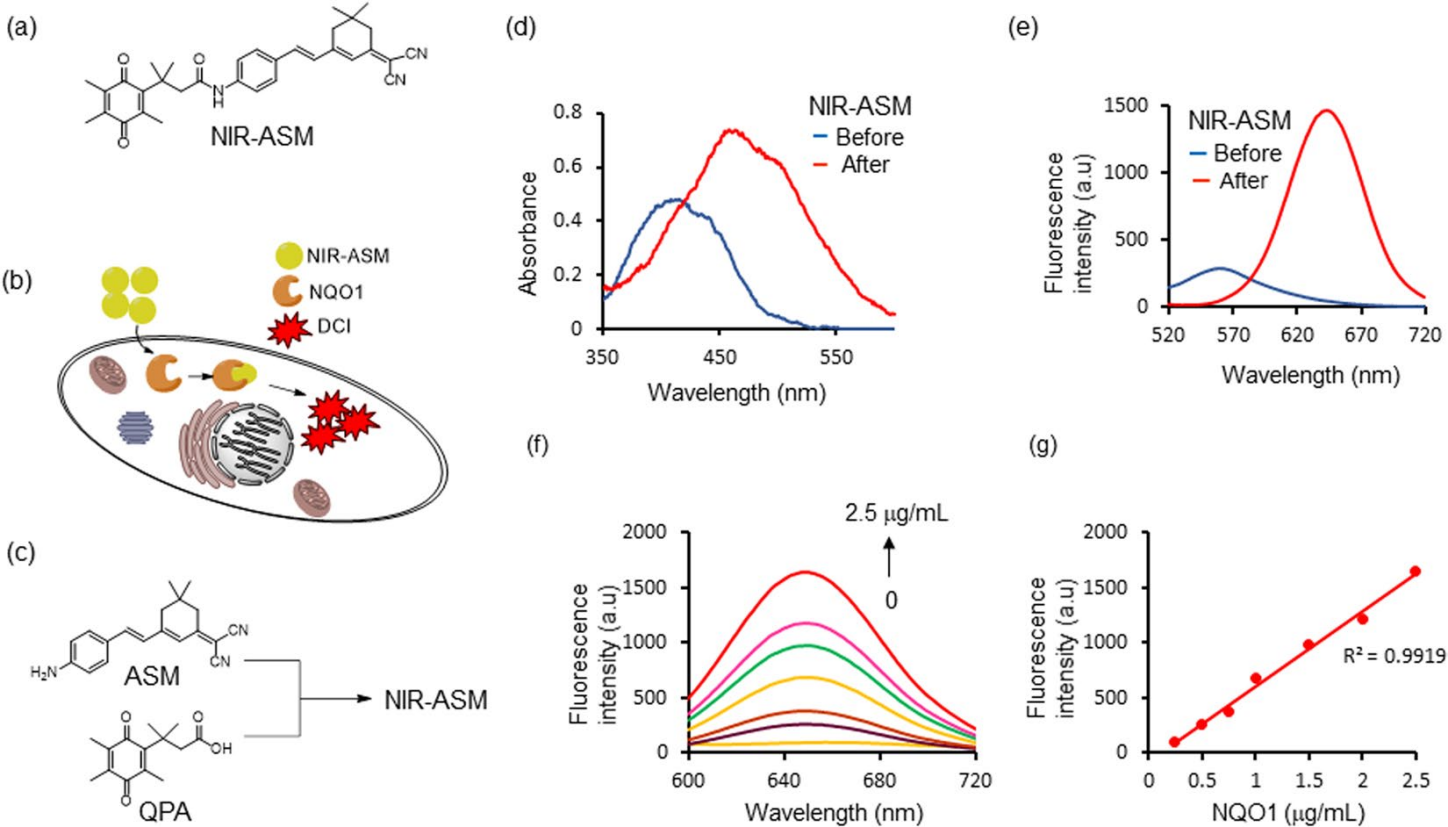
(**a**) The structure of NQO1 activatable NIR fluorescent probe NIR-ASM. (**b**) NIR-ASM activation and accumulation in NQO1 expressing cancer cells. (**c**) Synthesis of NIR-ASM by coupling ASM with quinone propionic acid (QPA) in the presence of EDCI. (**d**) absorbance and (**e**) fluorescence emission spectra of NIR-ASM before (blue line) and after (red line) upon activated by NQO1 in the presence of 100 µM NADH in PBS with 0.1% BSA (pH 7.4). (**f**) NQO1 concentration-dependent fluorescence (λ_max_ = 646 nm) spectra of NIR-ASM in the presence of 100 µM NADH. (**g**) The plot of fluorescence intensity versus NQO1 concentrations from 0.25 to 2.5 µg/mL. Experiments were performed in triplicate and averages were plotted.

Imaging agents emitting near-infrared fluorescence enable deeper penetration with low phototoxicity and high signal-to-background ratios due to minimal tissue auto-fluorescence, which is a prime requirement for *in vivo* imaging studies^40,41^. Accordingly, we designed an NQO1 activatable near-infrared fluorescent probe (NIR-ASM) by attaching a trimethyl-lock QPA with (E)-2-(3-(4-aminostyryl)-5,5-dimethylcyclohex-2-en-1-ylidene)malononitrile (ASM) for molecular imaging of cancer cells *in vivo* (Fig. 2a). The fluorophore ASM was used in this study because of its strong NIR fluorescence signal with substantial Stokes shift (~190 nm), to eliminate background interferences by avoiding reabsorption of emitted photons. We postulated that the initial fluorescence of NIR-ASM was significantly quenched due to the presence of QPA group capping at ASM via a stable amide bond, whereas NQO1 could cleave the amide bond and trigger the spontaneous elimination of dihydrocoumarin to liberate the ASM with remarkable NIR fluorescence enhancement. NQO1 is a ubiquitous cytosolic two-electron reductase that catalyzes the reduction of quinone substrates in the presence of NADH. The mechanism for visualizing NQO1 activity in living cells is shown in Fig. 2b. Upon interaction with NQO1 in the presence of NADH, QPA present in non-fluorescent NIR-ASM undergoes two-electron reduction to form an *o*-hydroxydihydrocinnamic acid derivative that undergoes rapid lactonization under physiological conditions to yield dihydrocoumarin, 6-hydroxy-4,4,5,7,8-pentamethylchroman-2-one (HPC) along with highly fluorescent ASM (Fig. S1). NQO1 catalyzed the hydrolysis of NIR-ASM in solution was monitored using HPLC analysis (Fig. S2) and results showed that NIR-ASM (HPLC retention time, *T*_*R*_ = 11.86 min) was nearly completely converted into ASM (*T*_*R*_ = 10.81 min) after 30 min of incubation along with the generation of HPC (*T*_*R*_ = 7.07 min). The synthesis of NIR-ASM was initiated from the preparation of fluorophore ASM and NQO1 substrate QPA according to the procedure outlined in Fig. S1 in the supporting information. ASM and QPA were coupled using EDCI as a catalyst and pyridine as a solvent (Fig. 2c). The chemical structures of these compounds were characterized by ^1^HNMR, ^13^CNMR and mass spectrometry. The purity of NIR-ASM was confirmed by HPLC.

### Fluorescent response of NIR-ASM towards NQO1

**Figure 3:**
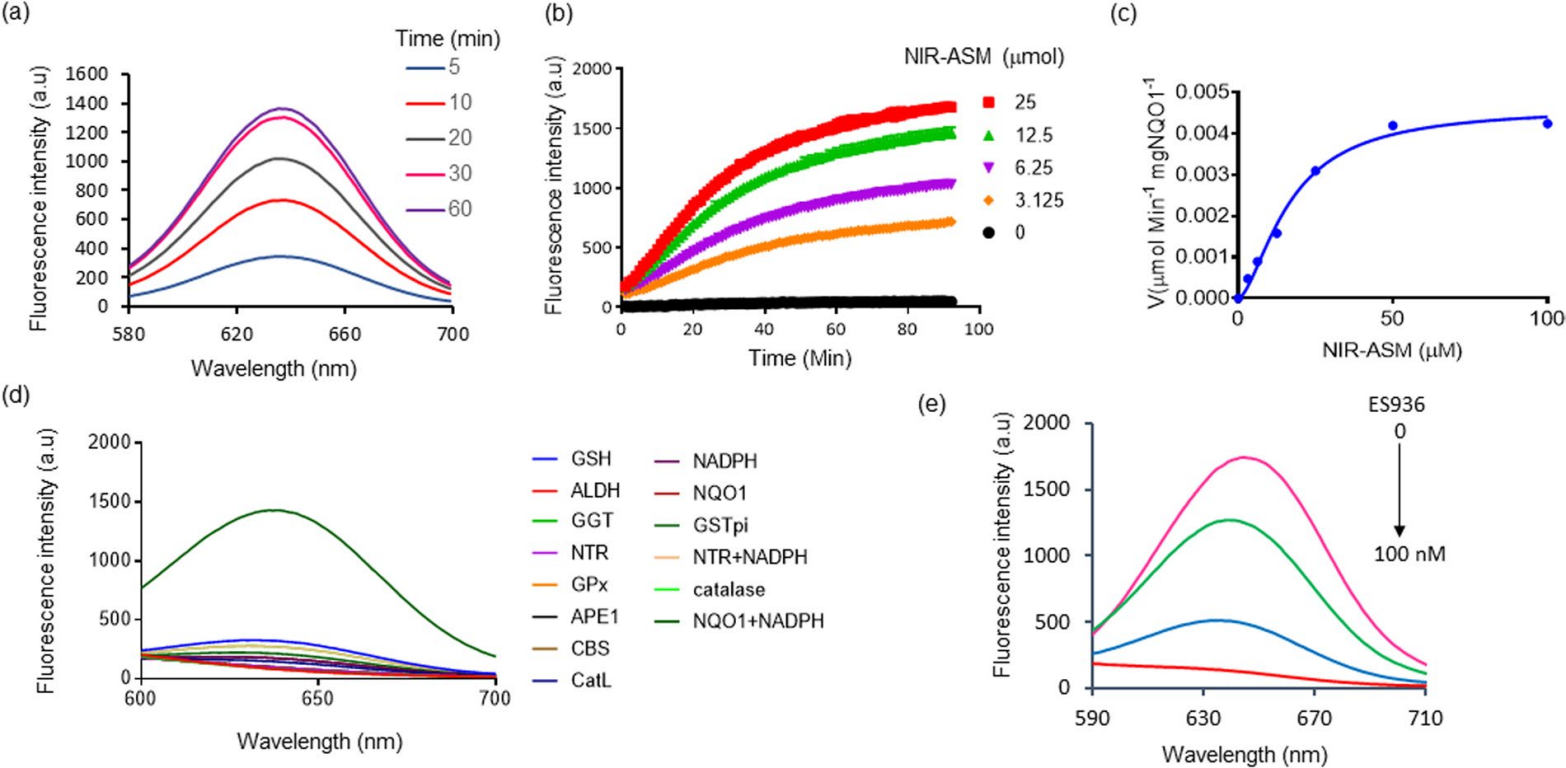
(**a**) Time-dependent NIR-ASM spectral changes initiated by the addition of NQO1 (2.5 µg/mL). (**b**) Time-dependent fluorescent intensity changes for the probe NIR-ASM in the presence of different concentrations of NQO1 (0 to 25 µM). Experiments were performed in triplicate and averages were plotted. (**c**) Kinetics plot of NQO1 (2 µg) towards NIR-ASM probe. Values shown are the average of 6 experiments. Assays were performed in 96 well plates at ex/em = 460/646 nm in the presence of 100 μM NADH. (**d**) Fluorescence response of NIR-ASM probe to various biologically relevant substrates. (**e**) Effect of NQO1 inhibitor ES936 on NQO1 activation of NIR-ASM. All experiments were conducted with 10 mM NIR-ASM and 100 mM NADH in 0.1% BSA containing PBS (pH 7.4) at λ_ex/em_ = 460/646 nm.

To unequivocally establish that NIR-ASM is a highly specific sensor for NQO1, we first investigated its spectral properties were systematically studied in simulated physiological conditions 0.1% BSA containing PBS, BSA used as a stabilizing agent, pH 7.4) at 37 °C, and the results are given in Fig. 2d,e. As shown in Fig. 2d, NIR-ASM displays one absorption band at 403 nm. However, upon addition of NQO1, the absorption at 403 nm disappears and a prominent absorption at 465 nm emerges; Along with the bathochromic shift of UV/Vis absorption, a distinct color change from yellow to red was observed after 30 min of incubation with NQO1. Further, the NIR-ASM itself showed no fluorescence emission in the NIR region due to the presence of an amine in the form of the amide. However, the reaction of the probe with NQO1 produced a strong fluorescence emission band at 646 nm with a large Stokes shift (186 nm) (Fig. 2f), which is highly advantageous for bioimaging analysis, because of non-overlapping excitation and emission wavelengths. Next, the fluorescence response of NIR-ASM to NQO1 at varying concentrations (0–2.5 μg/mL) was studied. As shown in Fig. 2f, the fluorescence intensity increased gradually with increasing the NQO1 concentration and reached a plateau at 2.5 μg/mL of NQO1. Also, the fluorescence enhancement was directly proportional to the enzyme concentration in the range of 0.125–2.5 μg/mL (R^2^ = 0.9919, Fig. 2g). The NQO1 detection limit (LOD) of NIR-ASM was calculated to be as low as 0.191 μg/mL, based on the standard deviation of the response (Sy) and the slope of the calibration curve (S) according to the formula: LOD = 3.3(Sy/S). Furthermore, in the presence of NQO1, NIR-ASM exhibited a time-dependent fluorescence enhancement (Fig. 3a) with the emission peak at 646 nm reaching a maximum after 30 min. Similar time-dependent fluorescence increase was observed with the different concentrations of NIR-ASM to NQO1 (Fig. 3b).

Next, the kinetics of fluorescent product formation was assessed. The incubation of NIR-ASM (0–25 μmol) with NQO1 (2 μg/mL) and its cofactor NADH (100 μM) exhibited a steady increase in the fluorescence suggesting a relatively fast rate of production of ASM (Fig. 3c). From this experiment, the initial rate of ASM formation, V (μmol min^−1^ μg NQO1^−1^), was calculated and then plotted as a function of NIR-ASM concentration (Fig. 3c). Apparent kinetic parameters were obtained by fitting the data in Fig. 3c to Michaelis-Menten kinetics, namely, the Michaelis constant (Km) = 33.82 ± 0.45 μM, maximum velocity (Vmax) = 0.00419 ± 0.00003 μmol min^−1^ mg NQO1^−1^, catalytic constant (kcat) = 13.01 min^−1^, and catalytic efficiency (kcat/Km) = 3.85 × 10^5^ M^−1^ min^−1^. Taken together, the data suggest NIR-ASM could be applied as a “turn-on” fluorescent sensor with a sensitive response to NQO1.

### Selectivity of NIR-ASM

The selectivity of NIR-ASM probe was studied by examining its reactivity towards various biomolecules and antioxidant enzymes such as the glutathione, aldehyde dehydrogenase 1, gamma-glutamyl transferase, glutathione peroxidase, apurinic/apyrimidinic endonuclease, cystathionine-β-synthase, cathepsin L, glutathione S-transferase, NADH alone and the combination of nitroreductase and NQO1. The probe NIR-ASM demonstrated high selectivity for NQO1 over the other enzymes/substrates tested, fluorescence resulted from the specific cleavage of QPA by NQO1 in the presence of its co-factor NADH (Fig. 3d). Next, the specificity for NQO1 was characterized by monitoring the fluorescence of NIR-ASM (10 mM) following incubation with NQO1, NQO1 pretreated with its inhibitor ES-926 (0–100 nM) a well-known NQO1 inhibitor^42^. As represented in Fig. 3e, ES936 effectively suppressed the fluorescence response in a concentration-dependent fashion, indicating the NQO1-dependent fluorescence response.

### Assessment of NIR-ASM biocompatibility

**Figure 4:**
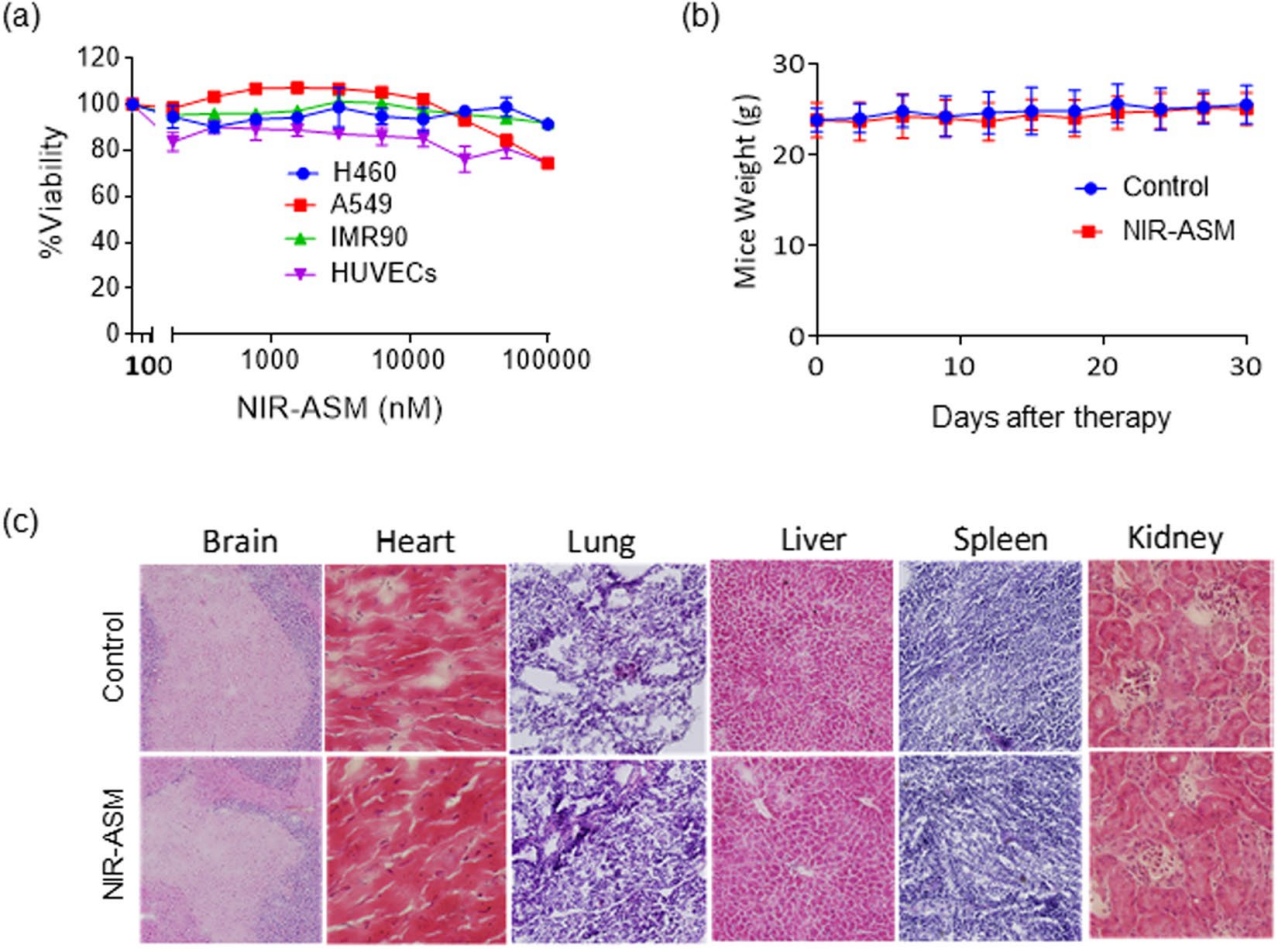
(**a**) Cytotoxicity of NIR-ASM against both cancer (A549 and H460) and normal (HUVECs and IMR90) cells. Cytotoxicity was analyzed by resazurin reduction assay by treating with 10 different concentrations of the NIR-ASM. (**b**) Animals were monitored for changes in body weight as a surrogate marker for toxicity after administration of 10 mg/Kg of NIR-ASM for 5 days a week for 4 weeks. (**c**) Lack of host toxicity as indicated by the H&E staining of major organs of mice treated with NIR-ASM compared to vehicle-treated (control) mice.

Inspired by the excellent photophysical properties of NIR-ASM in monitoring the NQO1 activity *in vitro*, we determined the biocompatibility of NIR-ASM both *in vitro* and *in vivo* before using for endogenous applications. The cytotoxicity of NIR-ASM was initially evaluated in cultured non-small-cell lung cancer cell lines A549 and NCI-H460, normal cells including lung fibroblasts (IMR 90) and human umbilical vein endothelial cells (HUVECs) to evaluate its biocompatibility using resazurin reduction assay^43^. The results revealed that NIR-ASM was not toxic to both normal and cancer cells even at higher concentrations (up to 100 µM) (Fig. 4a). Further, to evaluate NIR-ASM tolerability and toxicity *in vivo*, CD1 mice were grouped for the administration of vehicle (PEG:H_2_O: EtOH) alone or 5 mg/kg of NIR-ASM five times a week for five weeks. The average mouse weights were monitored as a surrogate marker to the toxicity. As shown in Fig. 4b, there were no remarkable changes in the average body weights, suggesting that the NIR-ASM treatment did not lead to overt toxicity of host tissues. Furthermore, there were no significant differences in the histological features among the NIR-ASM treatment and control groups in any of the tissues including the brain, heart, lung, liver, spleen, and kidney (Fig. 4c); these data indicate that NIR-ASM is unlikely to exert any adverse effects in the host at therapeutically relevant doses.

### Detection of NQO1 in live cells

**Figure 5:**
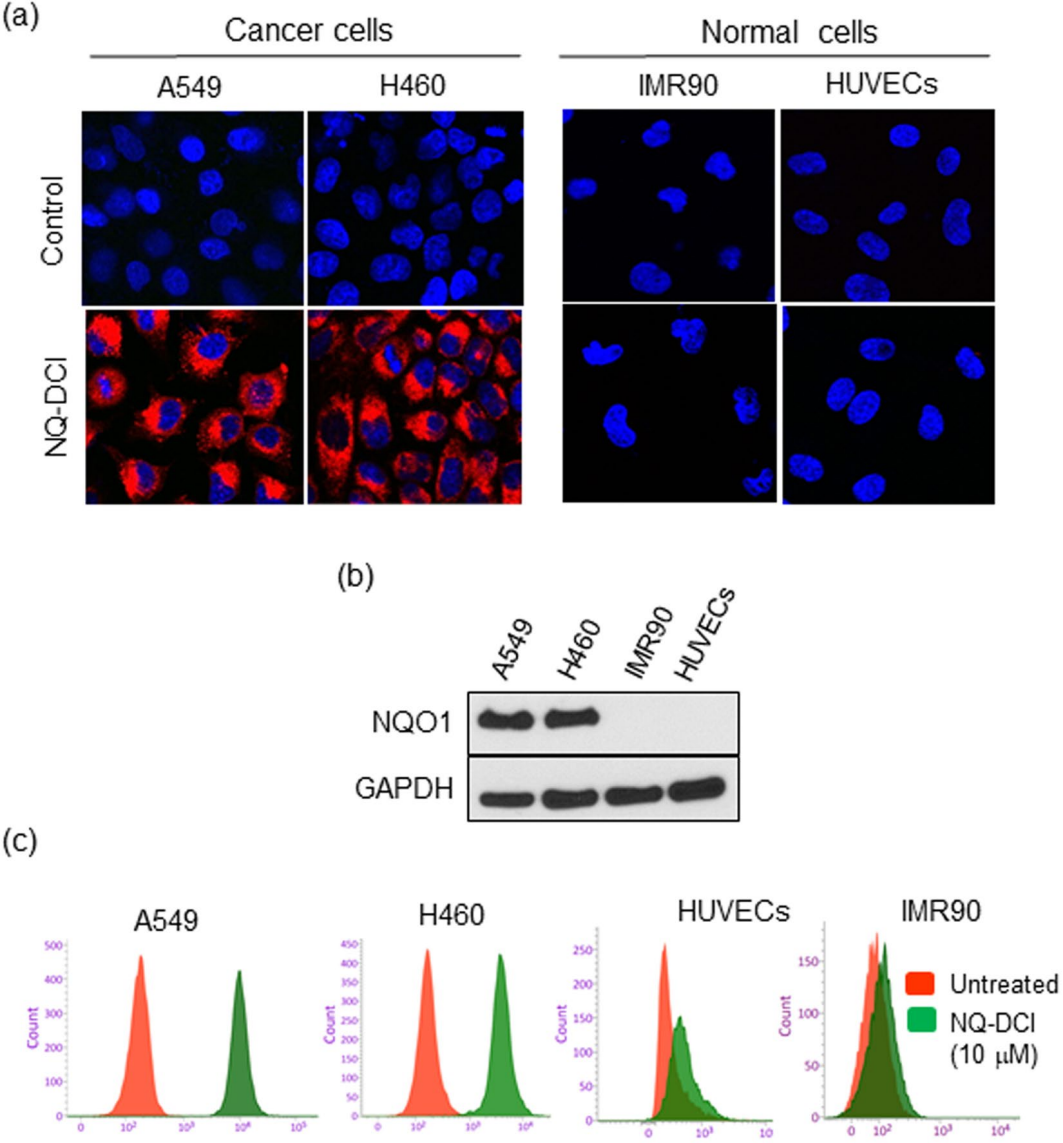
(**a**) Fluorescence images of NQO1-positive cancer cells NQO1-negative normal cells after incubation of 10 µM NIR-ASM for 1 h. The cell nuclei were counterstained by the Hoechst 33342 dye. (**b**) Western blot showing the expression of NQO1 in cancer cells in comparison to the normal cells, GAPDH was used as a loading control. (**c**) Flow cytometry assays were performed to determine NIR-ASM activation by NQO1-positive and negative cell lines. Assays were performed by incubating with 10 μM of NIR-ASM for 2 h and counting 1 × 10^4^ cells at λ_ex_ = 488 nm, λ_em_ = 617/40 nm.

Inspired by the excellent biocompatibility, we investigated its performance in both NQO1 expressing cancer cells (A549, H460) and NQO1 non-expressing normal cells (IMR90, HUVEC). All cells were incubated with NIR-ASM (10 μM) for 60 min, washed with PBS and were imaged under a confocal microscope after counterstaining the nuclei with Hoechst 33342 (Fig. 5a). The A549 and H460 cells produced a bright NIR fluorescence signal, whereas the normal cells did not show any fluorescence. The higher expression of NQO1 protein in A549 and H460 cells and its absence in normal cells, IMR90 and HUVEC as found with western blot analysis correlated with the probe reactivity in cells (Fig. 5b). These results further confirm the cell permeability of the NIR-ASM and its reaction with the intracellular NQO1 enzyme. Cell sorting using flow cytometry is a well-established technology in clinical diagnostics and biomedical research. To assess the applicability of the NIR-ASM to rapidly detect and quantify in both cancer and normal cells based on NQO1 expression, the probe was incubated with cell suspensions for 60 min, and about 1 × 10^4^ cells were analyzed on a flow cytometer. The resulting histograms for all the cell lines are shown in Fig. 5c. A high-intensity unimodal distribution of signals was obtained for NIR-ASM activation in each of the two NQO1-positive cancer cell lines (A549 and H460), while the negative normal cell lines IMR90 and HUVECs produced minimal fluorescence, thus confirming a rapid and detectable activation of the probe in A549 and H460 cells. Importantly, the sustained low fluorescence observed with the normal cells indicated the intracellular stability of NIR-ASM without any non-specific activation. These results clearly demonstrate that NIR-ASM has the ability to quantitatively detect endogenous NQO1 and can be used to rapidly differentiate tumor cells in fluidic streams.

### Chemical and genetic approaches confirm the specificity of NIR-ASM for NQO1 in live tumor cells

**Figure 6:**
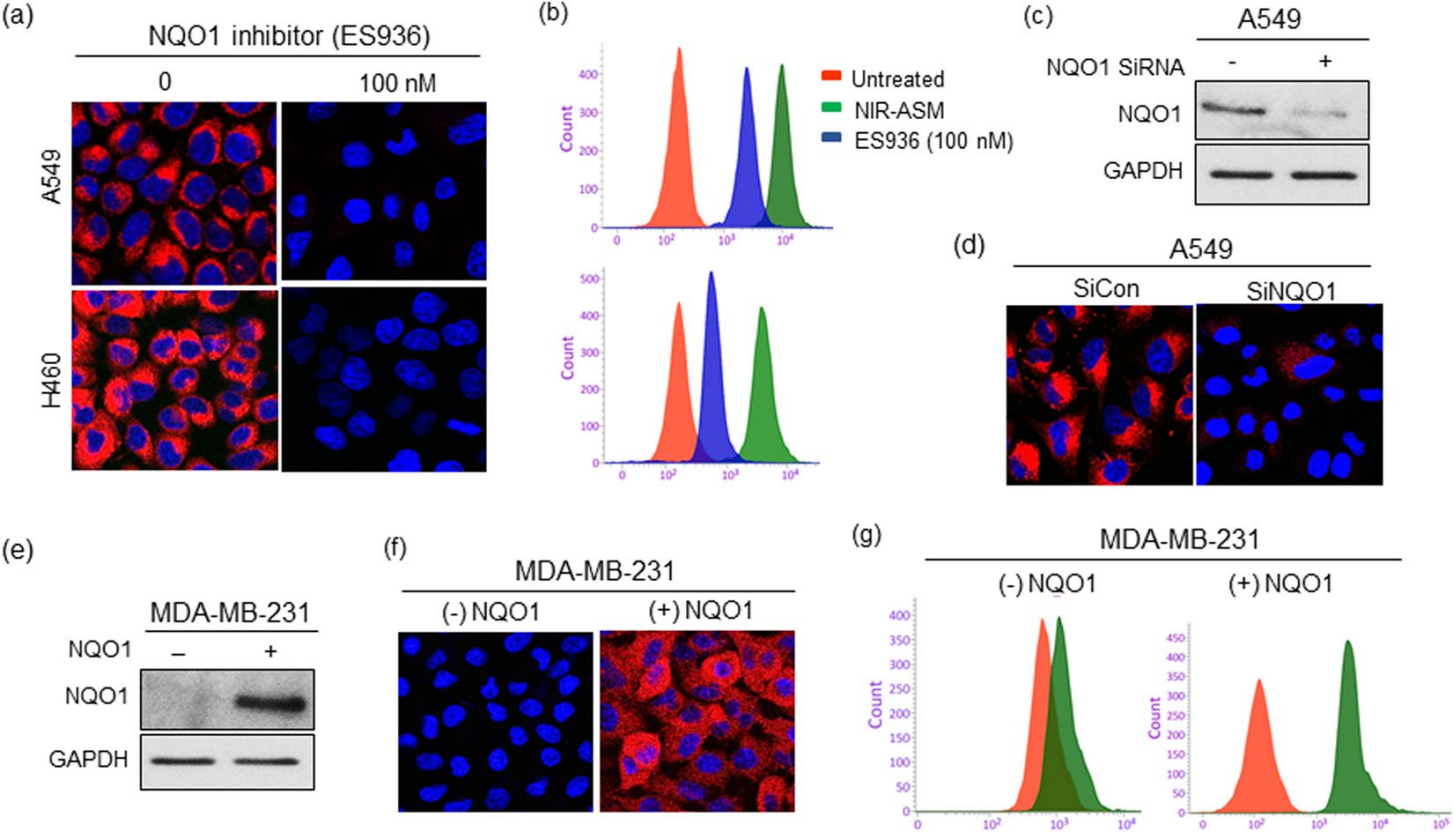
(**a**,**b**) Effect of NQO1 inhibitor ES936 (100 nM) on NIR-ASM fluorescence. Fluorescent microscopy (**a**) and flow cytometry (**b**) analysis of cells after treating with NIR-ASM (10 μM) for 1 h in the presence and absence of ES936 (100 nM). (**c**,**d**) Western blot showing the siRNA knockdown of NQO1 and its effect on the activation of NIR-ASM in A549 cells. (**e**) Western blot showing the transfection NQO1 into the NQO1 null MDA-MB-231 cells. (**f**,**g**) fluorescent microscopic images and flow cytometric analysis of wild-type (NQO1 negative) and NQO1 gene transfected (NQO1 positive) cells after incubating 10 µM NIR-ASM for 1 h.

A fluorescence probe with high sensitivity, simple operation, and excellent specificity will be an asset for the detection of NQO1 *in situ*. To assess the substrate selectivity of NIR-ASM for NQO1, both A549 and H460 cells were pretreated with 100 nM of NQO1 inhibitor ES936 for 6 h, before incubating with 10 μM NIR-ASM. ES936 completely blocked the NIR-ASM fluorescence when compared to the untreated cells (Fig. 6a). The effect of ES936 on endogenous NQO1 activity and subsequent reactivity with NIR-ASM was also determined by flow cytometry. The histograms in Fig. 6b, again show that the NIR-ASM induced fluorescence intensity was significantly decreased in ES936 pretreated tumor cells. In addition, as shown in Fig. 6c, a specific siRNA that silenced NQO1 gene expression more than 70% induced almost a total loss of NIR-ASM fluorescence compared with the control after 60 min incubation (Fig. 6d), suggesting that NQO1 was indeed responsible for the activation NIR-ASM fluorescent probe. To further demonstrate the difference in fluorescence signal observed in the cell images is certainly caused by NQO1 activity levels, we transfected the NQO1-negative MDAMB-231 breast cancer cells with an expression vector encoding a full-length human NQO1 protein followed by evaluation of NIR-ASM in parent and transfected cells. The presence of NQO1 protein in the transfected MDA-MB-231 cells and its absence in the untransfected control was confirmed by immunoblotting (Fig. 6e). Subsequently, the NQO1- negative and positive MDA-MB-231 cell lines were incubated with NIR-ASM and analyzed by fluorescence microscopy and flow cytometry for activation of the probe. No fluorescence was observed in control MDA-MB-231 cells, while intense fluorescence was evident after gene transfection (Fig. 6f,g). These data unambiguously confirm that the NIR-ASM probe is activated to the fluorescent ASM reporter depending on the levels of NQO1 protein present in tumor cells, thereby allowing accurate identification of NQO1-positive cells.

### In vivo real-time imaging of NQO1 in xenografts using NIR-ASM

**Figure 7:**
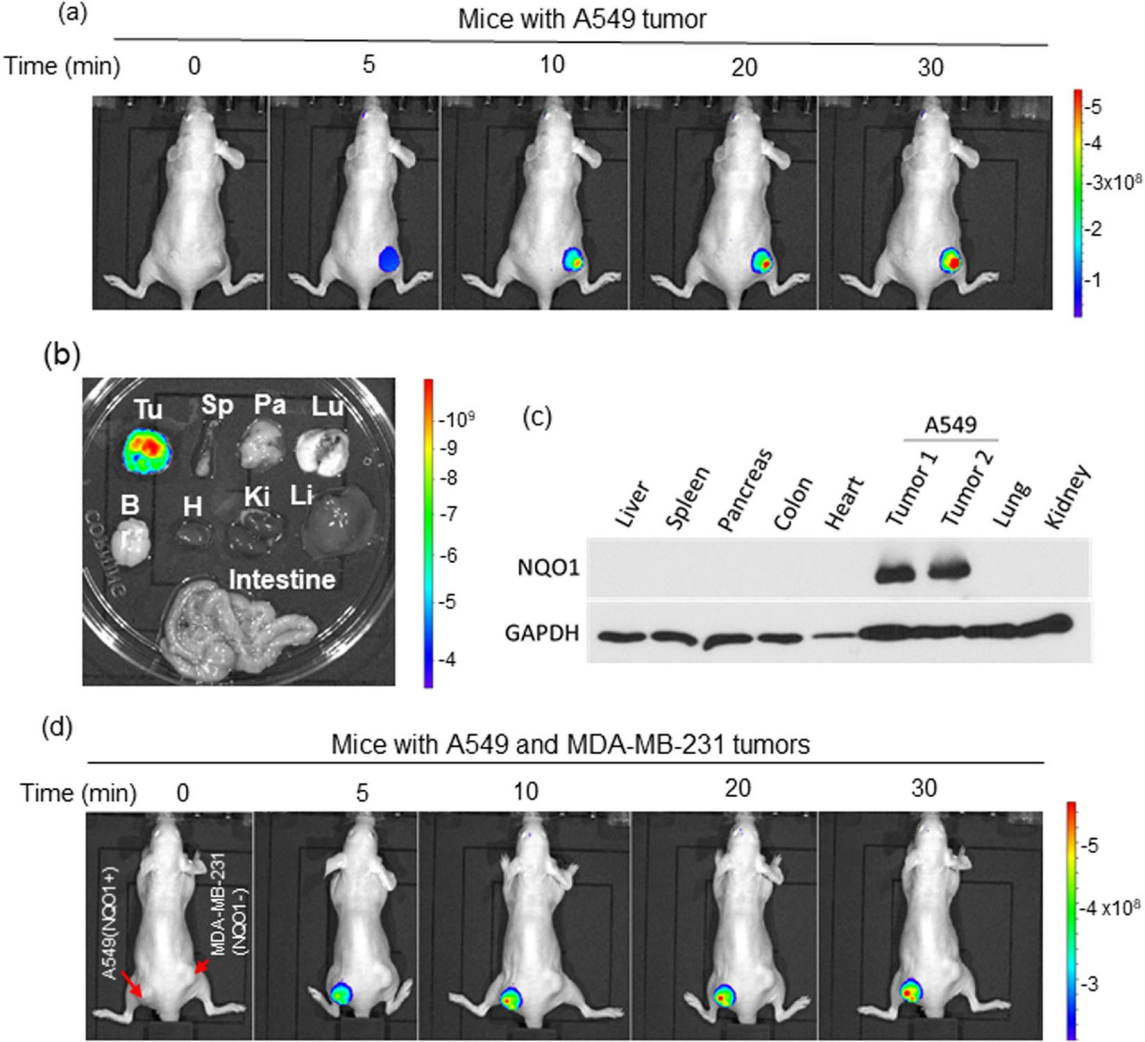
(**a**) Fluorescence imaging of endogenous NQO1 activity in A549 tumor-bearing nude mice after NIR-ASM (5 mg/kg, 50 µL) intravenous administration. (**b**) After 30 min of NIR-ASM administration, *ex vivo* fluorescent imaging of dissected organs such as the spleen (Sp), Pancreas (Pa), Lungs (Lu), Brain (Br), Heart (H), Kidneys (Ki), Liver (Li) and intestine along with tumor (Tu). (**c**) Immunoblot analysis showing the presence of NQO1 only in tumor lysates compared to other organs. (**d**) *In vivo* fluorescence imaging of nude mice bearing both NQO1 positive (A549) and NQO1 negative (MDA-MB-321) tumors after intravenous administration of NIR-ASM. *In vivo* imaging performed with IVIS Lumina XR Imaging system using excitation/emission filters of _~_500/640 nm. The scale indicates fluorescence intensity in terms of radiant efficiency.

*In vivo* real-time imaging offers a powerful tool for accurately diagnosing disease and suspicious lesions with valuable spatiotemporal precision. Having demonstrated the excellent specificity and performance of NIR-ASM in cultured cells, we explored its potential for real-time imaging of NQO1 activity in tumor-bearing mice. Tumor xenografts were established by implanting exponentially growing lung cancer cells (A549) subcutaneously into nude mice. When tumor growth reached the log phase, NIR-ASM (5 mg/kg in PEG:H_2_O: EtOH (6:3:1)) was given intravenously and subjected to whole-body fluorescence imaging using an *In Vivo* Imager (IVIS). As shown in Fig. 7a, a time-dependent and gradual increase in fluorescence signal in response to NQO1 was observed in the A549 tumor region and reached the plateau at 30 min. No signal was apparent in other organs of the animal. To confirm the tumor-selective targeting ability of NIR-ASM, the host organs and tumor from the mice administered with NIR-ASM were harvested and their fluorescence was analyzed *ex vivo* using the same IVIS. Fluorescent signals were observed only in the tumor and not in any other major organs including the lung, heart, spleen, kidney, liver, brain, pancreas and intestine (Fig. 7b). Furthermore, the lysates from the tumor and various organs were electrophoresed and western blotted using the NQO1 antibody. NQO1 protein at detectable levels was seen only in the tumor lysate and not in other tissues, thus, reinforcing the specificity of *in-vivo* imaging (Fig. 7c).

Additionally, to validate the NQO1-specific activation of NIR-ASM to generate fluorescence, we generated NQO1-positive (A549) and -negative (MDA-MB-231) xenografts in the same animal by injecting the corresponding tumor cells on the left and right flanks of the nude mice respectively. When tumors reached a volume of 200 mm^3^, NIR-ASM was administered through intravenous injection and the animals were imaged using the *In Vivo* imager. A gradual and productive fluorescence signal was observed only from the A549 tumor and no signal was discernible in the NQO1 negative MDAMB-231 tumors, again demonstrating the ability of our probe to distinguish between NQO1-positive and negative malignancies in real-time (Fig. 7d).

**Figure 8:**
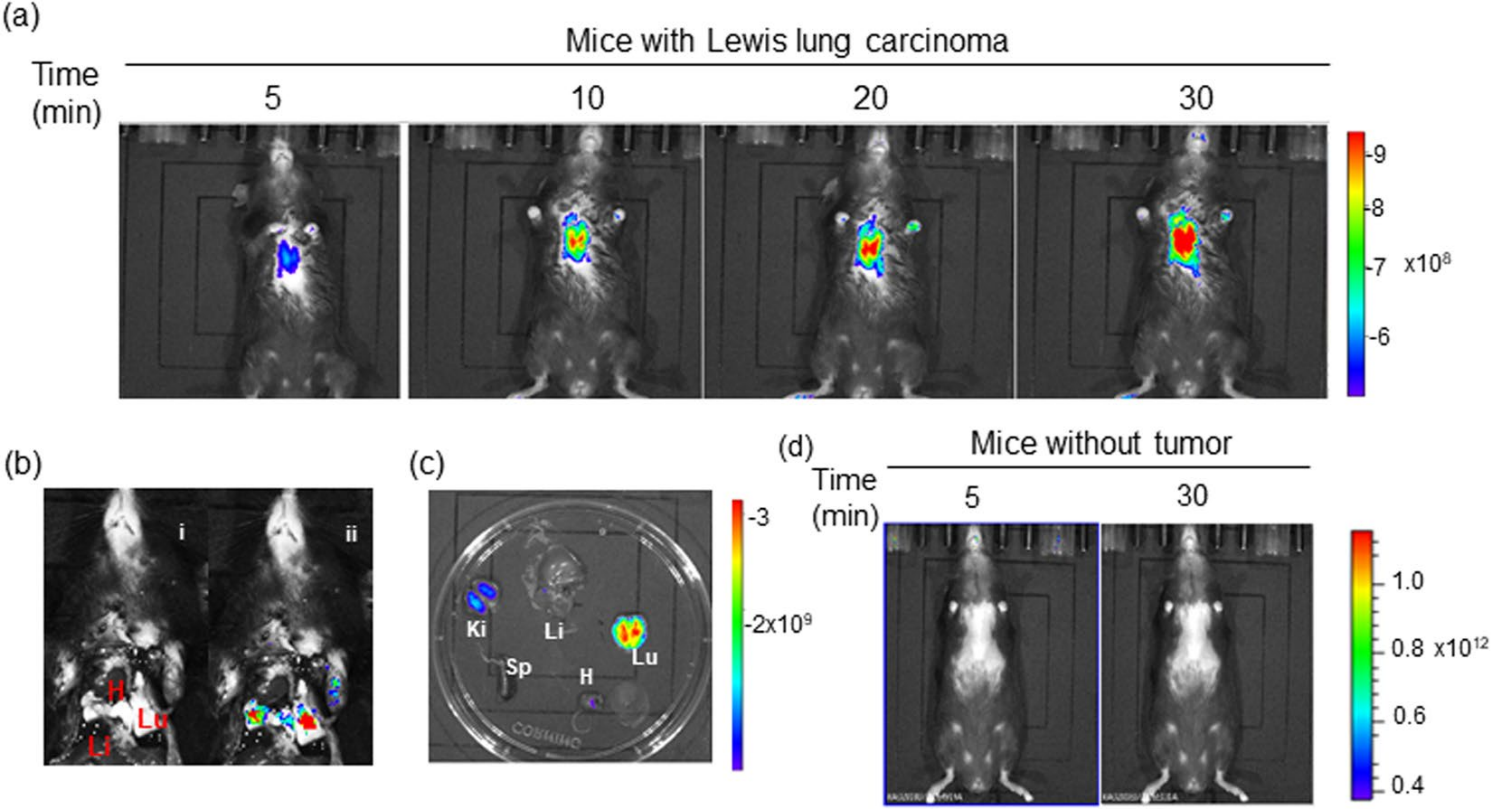
*In vivo* real-time fluorescence imaging in Lewis Lung Carcinoma (LLC) bearing BALB/c57 mice after intravenous administration of NIR-ASM (5 mg/kg, 50 µL). (**a**) time-dependent increase in the fluorescence intensity reaching a maximum at 30 min. (**b**) NIR-ASM illuminated lungs bearing LLC tumors with the minimal background; representative photograph (i) with markings of the heart (H), liver (Li) and Lungs (Lu) and composite image (ii) with fluorescence. (**c**) Fluorescent imaging of *ex vivo*-dissected organs such as the spleen (Sp), Lungs (Lu), Heart (H), Kidneys (Ki), Liver (Li) after administration NIR-ASM at 30 min. (**d**) *In vivo* fluorescence imaging of non–tumor-bearing mouse after intravenous administration of NIR-ASM. *In vivo* imaging performed with IVIS Lumina XR Imaging system using excitation/emission filters of ~500/640 nm. The scale indicates fluorescence intensity in terms of radiant efficiency.

Whether still *in-situ* or already disseminated, early diagnosis of cancer is an essential requirement for successful therapy. Although NQO1 is expressed at higher levels in cancers compared to a diminished or absent status in normal tissues, the diagnostic potential of existing NQO1 specific fluorescent probes has not been realized. Therefore, we evaluated the non-invasive diagnostic ability of NIR-ASM in a metastatic syngenic lung cancer model to mimic the clinical situation. Lung cancer was established in C57BL/6 mice by injecting Lewis Lung carcinoma (LLC) cells (0.5 × 10^6^ per animal) intravenously. This cell line is highly tumourigenic and is primarily used to model metastasis as well as evaluate the efficacy of chemotherapeutic agents *in vivo*^44^. The LLC cells have ~80 units of NQO1 activity compared to <10 units in healthy mouse lungs^45^. Two weeks after the implantation, the animals received NIR-ASM (5 mg/kg) intravenously. As demonstrated by both *in vivo* and *ex vivo* NIRF imaging (Fig. 8a,b), an intense fluorescent signal was observed from the tumor-bearing lungs, which was more than 100-fold higher NIRF signal intensity compared to other selected organs (Fig. 8c). Moreover, no fluorescence observed in the lungs of healthy mice after NIR-ASM administration (Fig. 8d). There are several routes for drug elimination from the body and the majority of drugs are eliminated by pathways that involve the kidneys. The fluorescence observed kidneys during *ex vivo* NIRF imaging indicating renal excretion might play an important role in eliminating NIR-ASM (Fig. 8c). In line with these data, we indicate that NIR-ASM fluorescent probe can be ideal for non-invasive diagnosis and early detection of NQO1 positive tumors *in vivo*.

In Summary, we have developed an enzyme-activatable, cell-permeable, non-toxic and biocompatible ‘turn-on’ NIR fluorescent probe (NIR-ASM) that provides accurate detection and visualization of endogenous NQO1 activity both *in vitro* and an *in vivo* preclinical model of lung and breast cancers. We demonstrated that NIR-ASM was highly stable and could be specifically activated by NQO1 to generate NIR fluorescence with a large Stokes shift (186 nm). NIR-ASM displayed a highly selective response toward NQO1 and did not generate fluorescence in other NADH requiring antioxidant reactions. The probe successfully differentiated NQO1 expressing cancer cells from normal cells and was validated using an NQO1 inhibitor, specific siRNA for NQO1 and by forced expression of the NQO1 protein. The NIR fluorescence of the probe showed a fast response and allowed a noninvasive *in vivo* real-time visualization of NQO1 in two subcutaneous and an orthotopic lung cancer-bearing mouse models without auto-fluorescence. The results suggest that NIR-ASM is an effective and promising NIR fluorescent probe that merits further development.

## Materials and Methods

All chemicals and solvents used in syntheses were purchased from Sigma- Aldrich or Fisher Scientific and used without further purification. NQO1 was purchased from Sigma-Aldrich (D1515). NQO1 siRNA (h) (sc-37139) and NQO1 (A180) (sc-32793) antibodies were purchased from Santa Cruz. All cell lines were obtained from ATCC (American type cell culture collection) The ^1^H, ^13^C NMR spectra were recorded on a Bruker-Avance 400 MHz Spectrometer. Chemical shifts (*δ*) are reported in ppm. ESI mass spectra were recorded on AB sciex QTRAP 5500 mass spectrometer. High-performance liquid chromatography (HPLC) was performed on an Agilent HPLC instrument. Peaks in NMR spectra are listed as singlet (s), doublet (d), triplet (t), or multiplet (m), and coupling constants (*J*) are reported in hertz (Hz). Fluorescence spectra were recorded on a Hitachi F-2500 Fluorescence spectrophotometer in a 10 mm standard cell with both excitation and emission slit widths of 10 nm. The incubation of NQO1 with NIR-ASM in the presence of NADH was carried out on a shaker at 37 °C. IVIS Lumina XR Imaging system (Caliper Life Sciences, Inc.) was used for the *in vivo* imaging. The BD LSRFortessa™ cell analyzer was used for the flow cytometric analysis. A Nikon multiphoton microscope equipped with NIS-Elements C acquisition and analysis software was used for the fluorescence microscopy.

### Spectroscopic measurements

NIR-ASM was dissolved with DMSO to prepare a stock solution, which was then diluted with enzyme reaction buffer (PBS containing 2.5 μg/mL, 100 μM NADH and 0.1% BSA) to prepare different concentrations of the probe for UV/Vis and fluorescence measurement. Time-dependent fluorescence intensity changes for different concentrations of NIR-ASM in the presence of NQO1 (2.5 μg/mL) and 100 μM NADH were measured using Clear bottom 96 well black plate and microplate reader. Fluorescence (ex. 460 nm and em. 646 nm) was quantitated every minute for 90 min with 20 seconds of shaking in between intervals.

### Kinetic studies

Kinetic studies were performed using a microplate reader under philological conditions (PBS with 0.1% of BSA at 37 °C). The assay buffer contained 2 µg/mL of NQO1, 100 µM NADH and different concentrations of NIR-ASM (0–100 µM) in a total volume of 100 µL. Time-dependent fluorescence measurements were carried out to monitor the release of ASM by NQO1 activation of NIR-ASM (λ_ex/em_ 460/646), and the data were collected every minute for 90 minutes. This assay was repeated two times in triplicate and the fluorescence readings were optimized to a standard curve of ASM of known concentration and calculated the velocity in terms of µmol^−1^min^−1^ µg NQO1. A plot of the velocities and NIR-ASM concentration was used to obtaining the apparent kinetic parameters Km and Vmax from nonlinear regression analysis and Michaelis-Menton constant using the GraphPad Prism.

### NQO1 inhibitor efficiency evaluation

NQO1 inhibitor efficiency was evaluated using specific NQO1 inhibitor ES936 under *in vitro* conditions. 2.5 μg/mL of NQO1 was incubated with different concentrations of ES936 (0–100 nM) in the presence of 100 μM NADH at 37 °C. Fluorescence emission was recorded after 30 min of incubation (λ_ex_. 460 nm).

### Cell culture

Non-small-cell lung cancer cell lines (A549 and H460) breast cancer cell line (MDA-MB-231) and human lung fibroblasts (IMR 90) were grown in Dulbecco’s modified Eagle’s (DMEM) medium supplemented with 10% Fetal Bovine Serum and 1% penicillin/streptomycin at 37 °C in a 5% CO_2_/95% air incubator. Primary human umbilical vein endothelial cells (HUVECs) were grown in M199 medium, supplemented with 15% fetal bovine serum, 150 mg/ml endothelial cell growth supplement, 5 U/ml heparin sodium and 1X antibiotic/antimycotic solution (Gibco).

### Knockdown of NQO1 by small interfering RNA (siRNA)

NQO1 and scrambled or control siRNAs were introduced into cells using the X-tremeGENE siRNA transfection reagent (Roche) according to the manufacturer’s instructions. 10 µl of transfection reagent was mixed with 80 pmol of siRNA and added to A549 cells in six-well plates. siRNA transfection efficiency was confirmed with Western blotting.

### NQO1 plasmid transfection

Cells were seeded at 60% confluence in six-well plates 16 h before transfection with the desired plasmids. Transfections were carried using a plasmid expressing wild-type NQO1 (pCDNA3 NQO1-Flag, addgene, 61729) and X-tremeGENE DNA transfection reagent (Roche) according to manufacturer instructions. One μg DNA was used in each transfection and cell extracts were prepared 24 h post-transfection to confirm NQO1 protein expression by Western blotting.

### Determination of NIR-ASM specificity towards NQO1

The specificity towards NQO1 was investigated by incubating NIR-ASM (10 μm) with various biologically relevant analytes such as glutathione (GSH, 1 mM), aldehyde dehydrogenase 1 (ALDH1A1, 2.5 μg/mL), gamma-glutamyl transferase (GGT, 10 U), glutathione peroxidase (GPx, 10 U), apurinic/apyrimidinic endonuclease (APE1, 10 U), cystathionine-β-synthase (CBS, 2 μg/mL), cathepsin L (CTSL, 2 μg/mL), glutathione S-transferase (GST-pi, 2 μg/mL), NADH (100 μM) alone and the combination of nitroreductase (NTR, 2 μg/mL) and NQO1 (2 μg/mL) for 30 min. Fluorescence spectra were analyzed to determine NIR-ASM specificity against NQO1.

### Cell viability assays

NIR-ASM was evaluated for *in vitro* cytotoxicity in human cancer (A549 and H460) and normal (IMR90 and HUVECs) cell lines following a protocol of 72 h continuous drug exposure using resazurin reduction assay. The cell lines were grown in 96-well microtiter plates at a density of 5000 cells per well. The plates were incubated for 24 h prior to addition of NIR-ASM. Ten concentrations of NIR-ASM were evaluated in quadruplicate sets. Plates were incubated for a further 72 h and replaced with 20 μL resazurin 0.01% (w/v) containing the medium. After 2 h, the fluorescence was measured using a Tecan Reader (Infinite m200 Pro) at a 544 nm excitation and 590 nm emission.

### Fluorescence imaging

Both cancer and normal cells were seeded into 6-well plates and cultured overnight in respective media at 37 °C. Cells were treated with 10 μM NIR-ASM and incubated at 37 °C for 60 min. They were then treated with nuclear stain Hoechst 33342 for 5 min. Finally, the medium was replaced with PBS and the live cells were imaged using a multiphoton confocal fluorescence microscope (λ_ex/em_ = 488/644 nm). For inhibitor study, cells were pretreated with NQO1 inhibitor ES936 (100 nM) for 6 h before adding the NIR-ASM.

### Flow cytometry analysis

The endogenous NQO1 activity of cancer cells and normal cells was determined by flow cytometry after NIR-ASM exposure. Cells were incubated with 10 μM of NIR-ASM for 60 min, harvested by trypsinization and washed with PBS. For inhibitor study, cells were pretreated with NQO1 inhibitor ES936 (100 nM) for 6 h before adding the NIR-ASM. About 1 × 10^4^ cells were analyzed by flow cytometry (λ_ex_ = 488 nm).

### Immunoblotting

Cells or tissues (100 mg/mL) were lysed in Cell Lysis Buffer (Cell Signaling, 9803) containing protease inhibitors and used for the immunoblotting. After estimating protein concentration using Bradford reagent (Bio-Rad, Hercules, CA), identical amounts of protein were fractionated by SDS-PAGE, and the proteins were electrophoretically-transferred to the polyvinylidene difluoride (PVDF) membranes. The hNQO1 monoclonal antibody was used for protein detection.

### Hematoxylin and eosin (H&E) staining

The hematoxylin and eosin staining were performed as described previously. Briefly, freshly dissected tissues were snap freeze and store at −20 °C for overnight. Frozen tissues were cut into 9 mm slices, the sections were stained in Mayer’s Hematoxylin and Eosin solution. Finally, the sections were dehydrated and mounted with Permount in a fume hood. The results were analyzed under a phase-contrast Olympus microscope (Olympus America Inc).

### *In vivo* bioimaging

The animal study protocol was approved by the Institutional Animal Use and Care Committee (IACUC), Texas Tech University Health Sciences Center, Protocol number: 07050. All experimental protocols used in this study were approved by IACUC, Texas Tech University Health Sciences Center and all the methods were carried out according to the committee guidelines. Female athymic nude mice (nu/nu, 4–6 weeks) were purchased from Charles River Laboratories (Wilmington, MA, USA). BALB/c57 mice were provided by Dr. Constantinos Mikelis of our University. To establish A549 or MDA-MB-231 xenografts, a total of 5 × 10^6^ cells (in 0.1 ml of serum-free media and Matrigel (1:1)) were subcutaneously injected into the flanks of the mice. For developing orthotopic lung cancer models, LLC cells (0.5 × 10^6^) were intravenously injected into the tail veins of female BALB/c57 mice. All animals were monitored for activity, physical condition, body weight, and tumor growth. To detect endogenous NQO1 activity, NIR-ASM was dissolved in a vehicle of composition, PEG-300: EtOH: saline (60:30:10, v/v/v) and given intravenously through tail vein injection. The animals were imaged at different time intervals with excitation/emission filters of ~500/640 nm using the IVIS Lumina XR Imaging system. At the end of the experiments, the xenografted tumors, hearts, lungs, livers, kidneys, spleens, and brains were removed and imaged to confirm the signal locations. Fluorescence represented in log values of radiance is photons per second per square centimeter per steradian.

## Supplementary information

Supplemental Information
