# Supplementary material for: Characterization of a highly specific NQO1-activated near-infrared fluorescent probe and its application for in vivo tumor imaging
Source: Sci Rep. 2019 Jun 12;9:8577. doi: 10.1038/s41598-019-44111-8 (PMC6562040; doi:10.1038/s41598-019-44111-8)
Supplement: Supplementary file 1 — Supplemental Information [file 41598_2019_44111_MOESM1_ESM.pdf]

Supporting information

Characterization of a highly specific NQO1-activated near-infrared fluorescent probe and its application for in vivo tumor imaging

Surendra Reddy Punganuru*[1], Hanumantha Rao Madala[1], Viswanath Arutla[1], Ruiwen Zhang[2] and Kalkunte S. Srivenugopal*[1]

[1]Department of Pharmaceutical Sciences, School of Pharmacy, Texas Tech University Health Sciences Center, Amarillo, TX 79106, USA.

[2]Department of Pharmacological and Pharmaceutical Sciences, College of Pharmacy, University of Houston, Houston, TX 77204, USA.

*Corresponding Authors: Kalkunte S. Srivenugopal, Phone: 806-414-9212; E-mail: Kalkunte.Srivenugopal@ttuhsc.edu; Surendra R. Punganuru, Phone: 806-414-9224; E-mail: Surendra.r.punganuru@ttuhsc.edu

Contents

1. Materials and Methods S2
2. Synthesis of NQ-DCI (Figure S1) and procedures S2-7
3. Figure S2. HPLC analysis of NQ-DCI activation S8
4. Original images of western blots on the film S9-15
5. Figures S5-S16. ^{1}H and ^{13}C spectra of NQ-DCI and intermediates S16-27

Materials and Methods. All chemicals and solvents used in syntheses were purchased from Sigma- Aldrich or Fisher Scientific and used without further purification. Galss TLC silica gel 60 F254 were purchased from EMD Millipore and used to perform thin layer chromatography. SilicaFlash P60 (230-400 mesh) purchased from Silicycle was used for the purification of products. The ^{1}H, ^{13}C NMR spectra were recorded on a Bruker-Avance 400 MHz Spectrometer. Chemical shifts (*δ*) are reported in ppm. ESI mass spectra was recorded on AB sciex QTRAP 5500 mass spectrometer. High-performance liquid chromatography (HPLC) was performed on an Agilent HPLC instrument. Peaks in NMR spectra are listed as singlet (s), doublet (d), triplet (t), or multiplet (m), and coupling constants (*J*) are reported in hertz (Hz).

Synthesis of NQ-DCI

NQ-DCI was prepared as described in scheme 1.

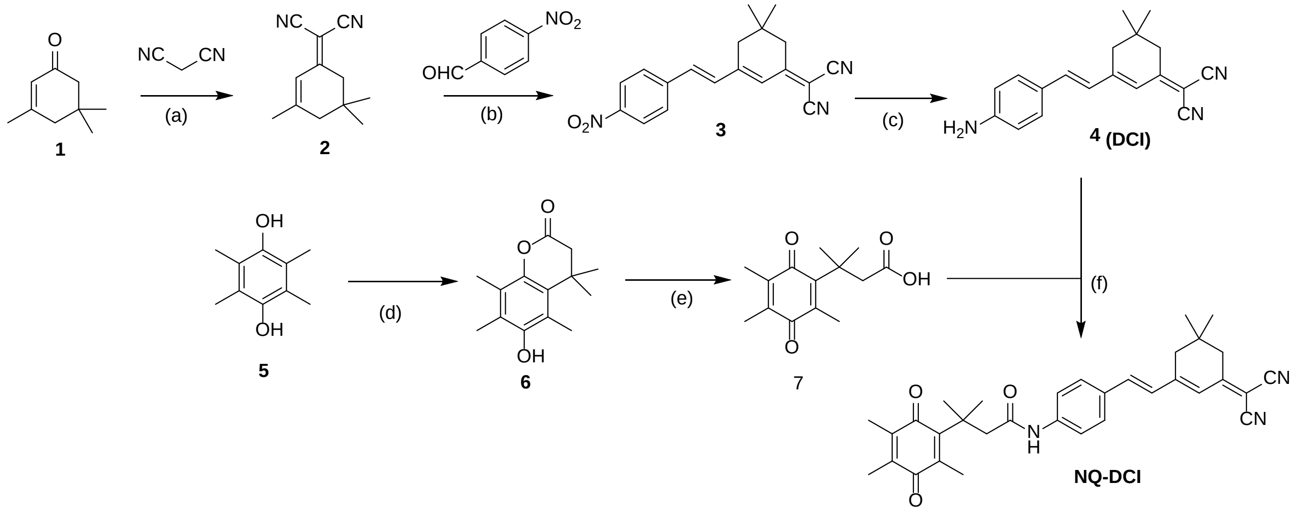

Figure S1.

Reagents & conditions: (a) Ammonium acetate, acetic anhydride, acetic acid, toluene, reflux, 12 h; (b) piperidine, acetonitrile, 50 ^{o}C, 6 h; (c) $SnCl_2$, HCl, ethyl acetate, 8 h; (d) methyl 3,3-dimethylacrylate, methanesulfonic acid, 70 ^{o}C, 2 h; (e) NBS, acetonitrile, water, rt, 1 h; (f) EDC, pyridine, rt, 10 h.

Synthesis of Quinone propionic acid (QPA) (7): QPA was prepared from commercially available stating materials 2,3,5,6-tetramethylbenzene-1,4-diol (5) and methyl 3,3-dimethylacrylate using methane sulfonic acid as a catalyst as presented in scheme 1.

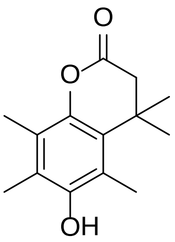

Synthesis of 6-hydroxy-4,4,5,7,8-pentamethylchroman-2-one (6): To a stirred solution of 2.25 g (13.5 mmol) of 2,3,5,6-tetramethylbenzene-1,4-diol (5) in 15 mL of methanesulfonic acid was added 1.98 mL (1.85 g, 16.2 mmol) of methyl 3,3-dimethylacrylate at room temperature. The reaction mixture was stirred at 70 °C for 2 h. After cooling to room temperature, the reaction mixture was diluted with 150 mL of water and extracted three 70 mL portions of dichloromethane. The combined organic extracts were washed with 100 mL of saturated $NaHCO_3$ solution, 100 mL of NaCl solution, dried over anhydrous Na_2SO_4 and concentrated under diminished pressure. The crude product was recrystallization from 30% $CHCl_3$ in hexanes gave compound 6 as white solid: yield 2.56 g (81%); ^1H NMR (400 MHz, $CDCl_3$) δ 1.45 (s, 6H), 2.19 (s, 3H), 2.22 (s, 3H), 2.36 (s, 3H), 2.55 (s, 2H) and 4.75 (s, 1H); ^{13}C NMR (100 MHz, $CDCl_3$) δ 12.3, 12.6, 14.5, 27.7, 35.5, 46.1, 118.9, 121.8, 123.4, 128.2, 143.5, 148.8 and 168.9. ESI MS (*m/z*): 234 (M^+)

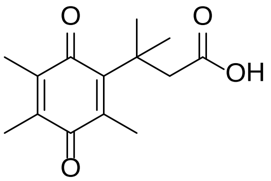

Synthesis of 3-methyl-3-(2,4,5-trimethyl-3,6-dioxocyclohexa-1,4-dien-1-yl)butanoic acid (7, QPA) (7): To a stirred solution of 2.00 g (8.54 mmol) of compound 6 in a solution of 70 mL of acetonitrile and 30 mL of water was added 1.67 g (9.39 mmol) of *N*-bromosuccinimide at room temperature. After 60 min, acetonitrile was removed from the reaction mixture, the residue was extracted three 30-mL portions of dichloromethane. The combined organic layer was dried over anhydrous Na_2SO_4 and concentrated under diminished pressure. The crude product was purified by chromatography on a silica gel column. Elution with 4:1 hexanes-ethyl acetate afforded

compound 7 as yellow solid: yield 1.64 g (77%); silica gel TLC R_f 0.23 (7:3 hexanes/ethyl acetate); ^{1}H NMR (400 MHz, $CDCl_3$) δ 1.43 (s, 6H), 1.92 (s, 3H), 1.95 (s, 3H), 2.13 (s, 3H) and 3.01 (s, 2H); ^{13}C NMR (100 MHz, $CDCl_3$) δ 12.2, 12.6, 14.4, 29.0, 38.1, 47.4, 138.5, 139.2, 143.1, 152.1, 178.6, 187.6 and 191.0. ESI MS (*m/z*): 249.1 (M-1).

Synthesis of DCI

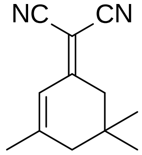

2-(3,5,5-trimethylcyclohex-2-en-1-ylidene) malononitrile (2): To a stirred solution of 2.25 mL (15.0 mmol) isophorone (**1**) and 1.49 g (22.5 mmol) of malononitrile in 50 mL toluene were added 0.29 g (3.75 mmol) of ammonium acetate, 0.5 mL of glacial acetic acid, and 1.0 mL of acetic anhydride. The reaction mixture was heated at 120 ^oC for 12 h under air atmosphere in the dark. Reaction mixture was allowing to cool to room temperature, solvent was removed under diminished pressure, neutralized with saturated sodium carbonate solution and extracted with three 30 mL portion of ethyl acetate. The combined organic layer was dried over anhydrous Na_2CO_3, and concentrated. The crude product was purified by chromatography on a silica gel column. Elution with 9:1 hexanes-ethyl acetate afforded compound **2** as pale yellow solid: yield 1.98 g (71%); silica gel TLC R_f 0.37 (4:1 hexanes/ethyl acetate); ^{1}H NMR (400 MHz, $CDCl_3$) δ 1.01 (s, 6H), 2.03 (s, 3H), 2.18 (s, 2H), 2.51 (s, 2H) and 6.62 (s, 1H); ^{13}C NMR (100 MHz, $CDCl_3$) δ 25.3, 27.8, 32.4, 42.6, 45.6, 78.2, 112.4, 113.2, 120.5, 159.9 and 170.4. ESI MS (*m/z*): 186.1 (M^+)

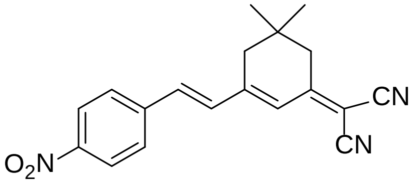

(*E*)-2-(5,5-dimethyl-3-(4-nitrostyryl) cyclohex-2-en-1-ylidene) malononitrile (3): To a stirred solution of 1.92 g (10.3 mmol) of compound **2**, 0.15 mL of piperidine in 40 mL of acetonitrile was added 4-nitrobenzaldehyde (2.34 g, 15.5 mmol). The reaction mixture was stirred at 50 ^oC for 6 h. After the reaction mixture was allowed to cool to room temperature, the formed precipitate was filtered, washed with acetonitrile, and recrystallized with ethanol to obtained compound **3**

as yellow crystal: yield 2.96 g (90%); ^{1}H NMR (400 MHz, $CDCl_3$) δ 1.11 (s, 6H), 2.50 (s, 2H), 2.64 (s, 2H), 6.94 (s, 1H), 7.06 (d, 1H, J = 16.1 Hz), 7.13 (d, 1H, J = 16.2 Hz), 7.66 (d, 2H, J = 8.8 Hz) and 8.25 (d, 2H, J = 8.7 Hz); ^{13}C NMR (100 MHz, $CDCl_3$) δ 28.0, 32.0, 39.1, 42.9, 80.7, 112.3, 113.0, 124.3, 125.6, 127.9, 133.2, 133.7, 141.9, 147.9, 152.2 and 168.8. ESI MS (*m/z*): 319.2 (M^+)

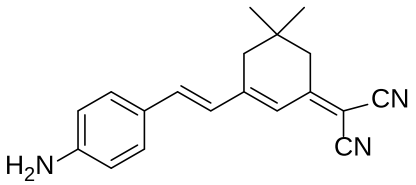

(*E*)-2-(3-(4-aminostyryl)-5,5-dimethylcyclohex-2-en-1-ylidene) malononitrile (4): To a stirred solution of 2.60 g (8.14 mmol) of compound **3** and 2.32 g (12.2 mmol) of $SnCl_2$ in 50 mL of ethyl acetate was added 0.5 mL of hydrochloric acid dropwise. The reaction mixture was refluxed for 8 h. After, the solvent was evaporated under reduced pressure, the obtained residue was washed with 15% sodium hydroxide solution and extracted with three 50 mL portions of ethyl acetate. The combined organic layer was dried over anhydrous Na_2SO_4, and concentrated. The resulted crude product was further purified by chromatography on a silica gel column. Elution with 4:1 hexanes-ethyl acetate afford compound **4** as red powder: yield 2.07 g (88%); silica gel TLC R_f 0.18 (7:3 hexanes/ethyl acetate); ^{1}H NMR (400 MHz, $CDCl_3$) δ 1.07 (s, 6H), 2.44 (s, 2H), 2.58 (s, 2H), 3.99 (brs, 2H), 6.67 (d, 2H, J = 8.5 Hz), 6.76 (s, 1H), 6.81 (d, 1H, J = 16.0 Hz), 7.00 (d, 1H, J = 16.0 Hz) and 7.34 (d, 2H, J = 8.4 Hz); ^{13}C NMR (100 MHz, $CDCl_3$) δ 28.0, 32.0, 39.2, 43.0, 113.2, 114.0, 115.1, 121.9, 125.3, 126.0, 129.5, 137.7, 148.4, 154.9 and 169.3. ESI MS (*m/z*): 288.1 (M-1)

Synthesis of NQ-DCI: NQ-DCI was synthesized by coupling (*E*)-2-(3-(4-aminostyryl)-5,5-dimethylcyclohex-2-en-1-ylidene) malononitrile (4, DCI) and 3-methyl-3-(2,4,5-trimethyl-3,6-dioxocyclohexa-1,4-dien-1-yl) butanoic acid (7, QPA) using 1-Ethyl-3-(3-dimethylaminopropyl) carbodiimide (EDC).

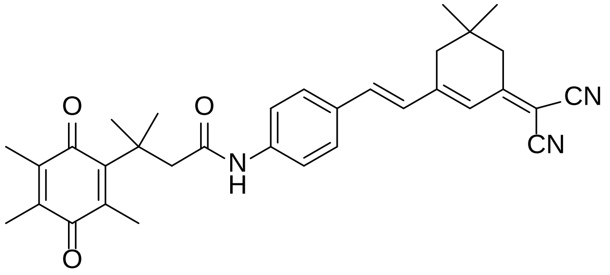

(*E*)-*N*-(4-(2-(3-(dicyanomethylene)-5,5-dimethylcyclohex-1-en-1-yl)vinyl)phenyl)-3-methyl-3-(2,4,5-trimethyl-3,6-dioxocyclohexa-1,4-dien-1-yl)butanamide (NQ-DCI): To a stirred solution of 250 mg (1.00 mmol) of compound **7** in 1 mL of dry pyridine was added 287 mg (1.50 mmol) of EDC at room temperature. After 20 min a solution of 289 mg (1.00 mmol) of compound **4** in 0.2 mL of pyridine was added to the reaction mixture and stirred for 10 h at room temperature. The reaction mixture was diluted with 50 mL of 1N HCl and extracted with two 30 mL portions of ethyl acetate. Combined organic layer was dried over Na_2SO_4 and concentrated under diminished pressure. The obtained crude product was purified by chromatography on a silica gel column. Elution with 4:1 hexanes-ethyl acetate afforded compound **NQ-DCI** as yellow solid: yield 427 mg (82%); silica gel TLC R_f 0.19 (7:3 hexanes/ethyl acetate); ^{1}H NMR (400 MHz, $CDCl_3$) δ 1.07 (s, 6H), 1.50 (s, 6H), 1.95 (s, 3H), 1.96 (s, 3H), 2.16 (s, 3H), 2.45 (s, 2H), 2.59 (s, 2H), 3.06 (s, 2H), 6.81 (s, 1H), 6.89 (d, 1H, J = 16.1 Hz), 6.99 (d, 1H, J = 16.1 Hz), 7.34 (s, 1H), 7.43 (d, 2H, J = 8.8 Hz) and 7.47 (d, 2H, J = 8.7 Hz); ^{13}C NMR (100 MHz, $CDCl_3$) δ 12.2, 12.7, 14.2, 28.0, 29.1, 32.0, 38.4, 39.2, 43.0, 50.5, 78.3, 112.8, 113.6, 119.8, 123.3, 128.2, 128.4, 131.5, 136.4, 138.4, 138.5, 139.0, 143.2, 152.7, 154.0, 169.3, 170.4, 187.5 and 191.5. ESI MS (*m/z*): 521.3 (M-1).

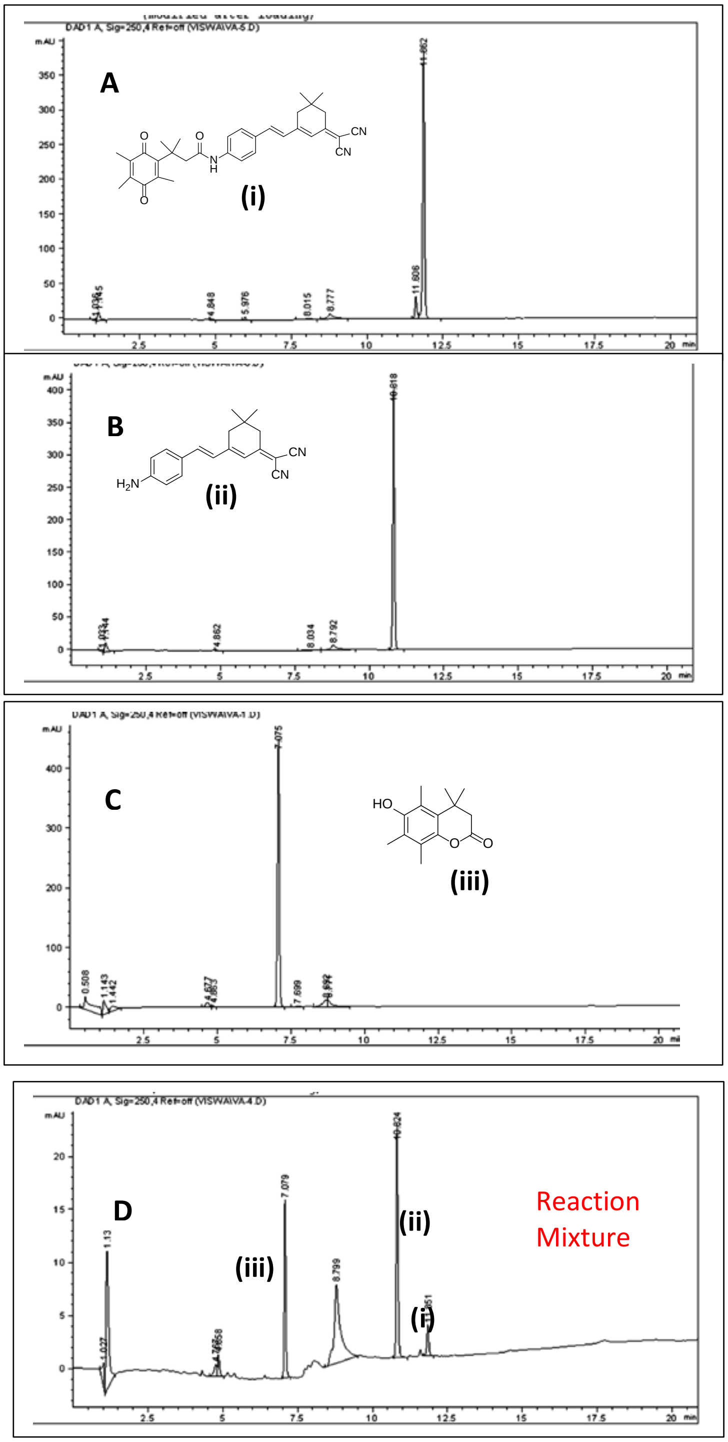

Figure S2. HPLC analysis of NQ-DCI activation. (A) HPLC spectrum of NQ-DCI (B) HPLC spectrum of DCI (c) dihydrocoumarin (HPC) (D) HPLC spectrum of reaction mixture having 10 μM NQ-DCI, 2.5 mg/mL NQO1 and 100 μM NADPH.

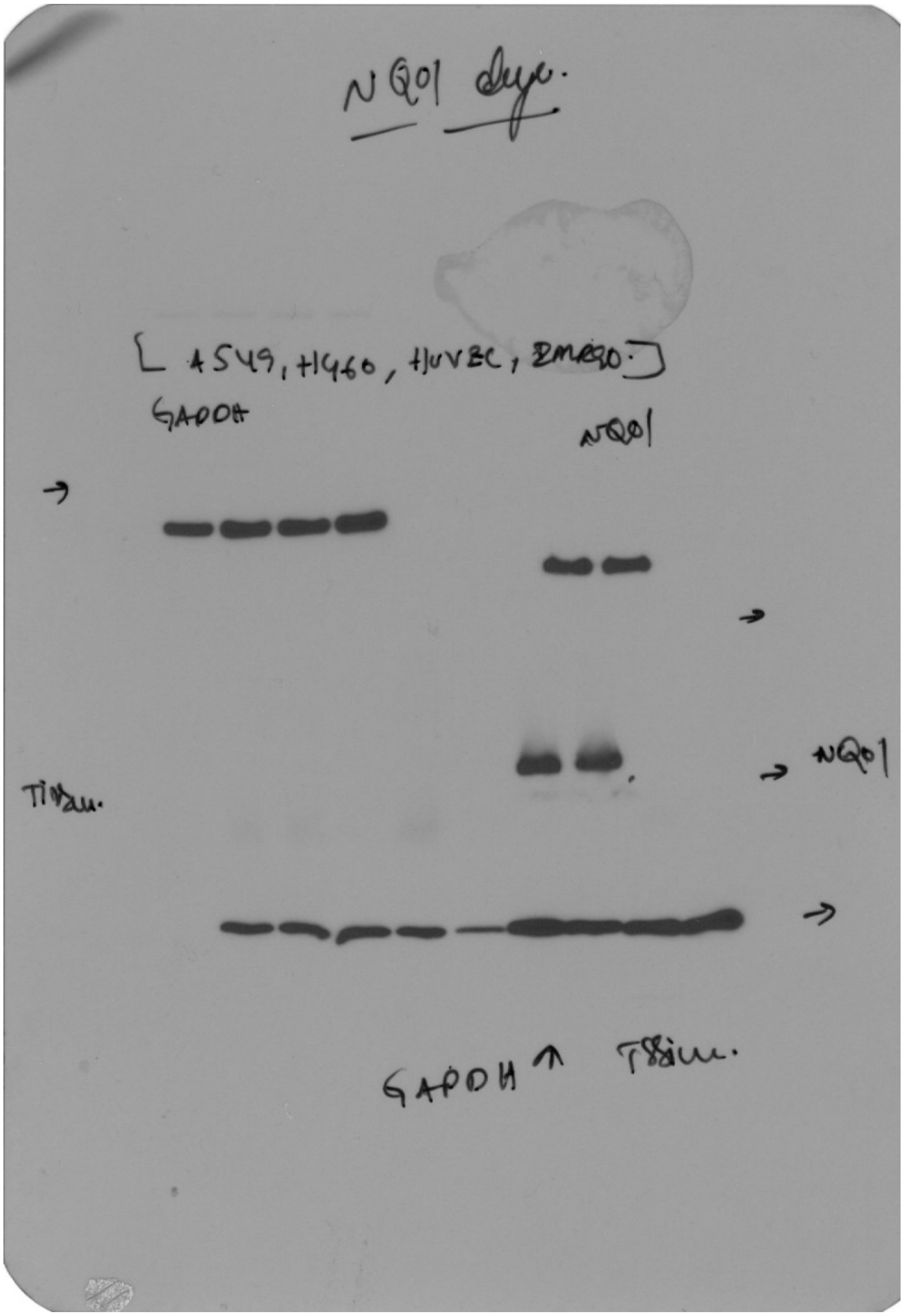

Figure S6. Film Showing Western blots presented in the manuscript (Version 1: Low exposure; 30 Sec)

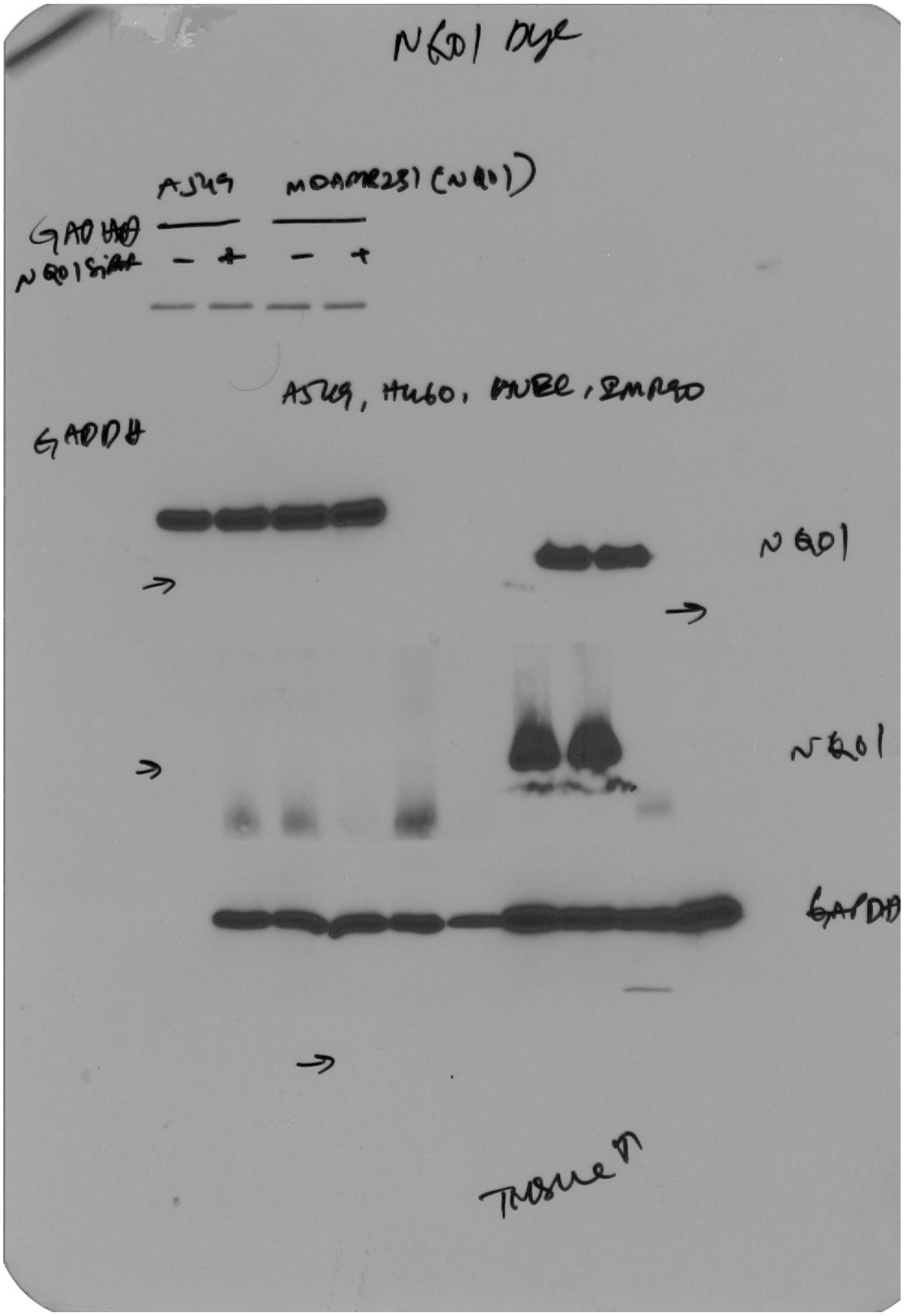

Figure S7. Film Showing Western blots presented in the manuscript (Version 2: long exposure; 1min)

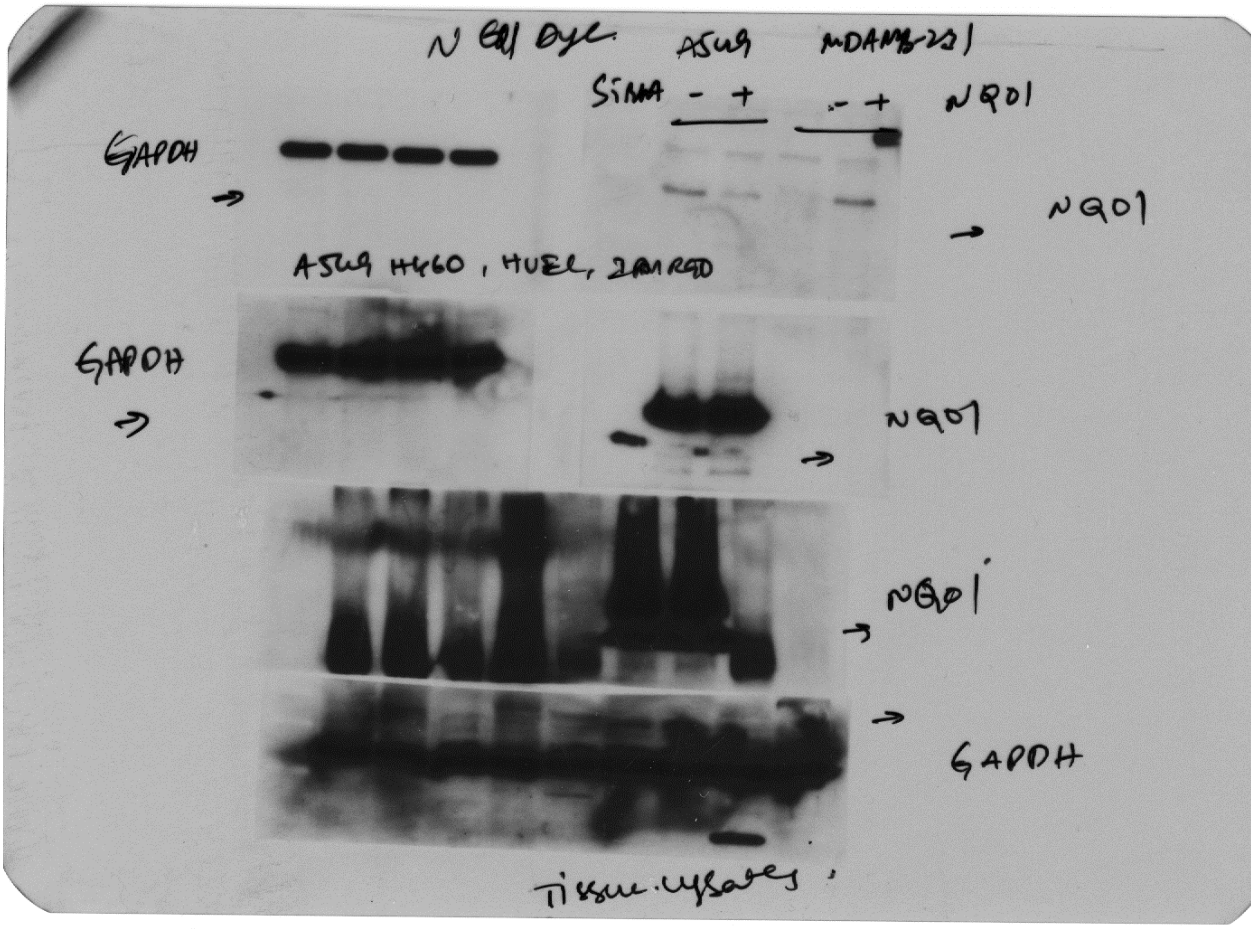

Figure S8. Film Showing Western blots presented in the manuscript (Version 3: Longest exposure; 2min)

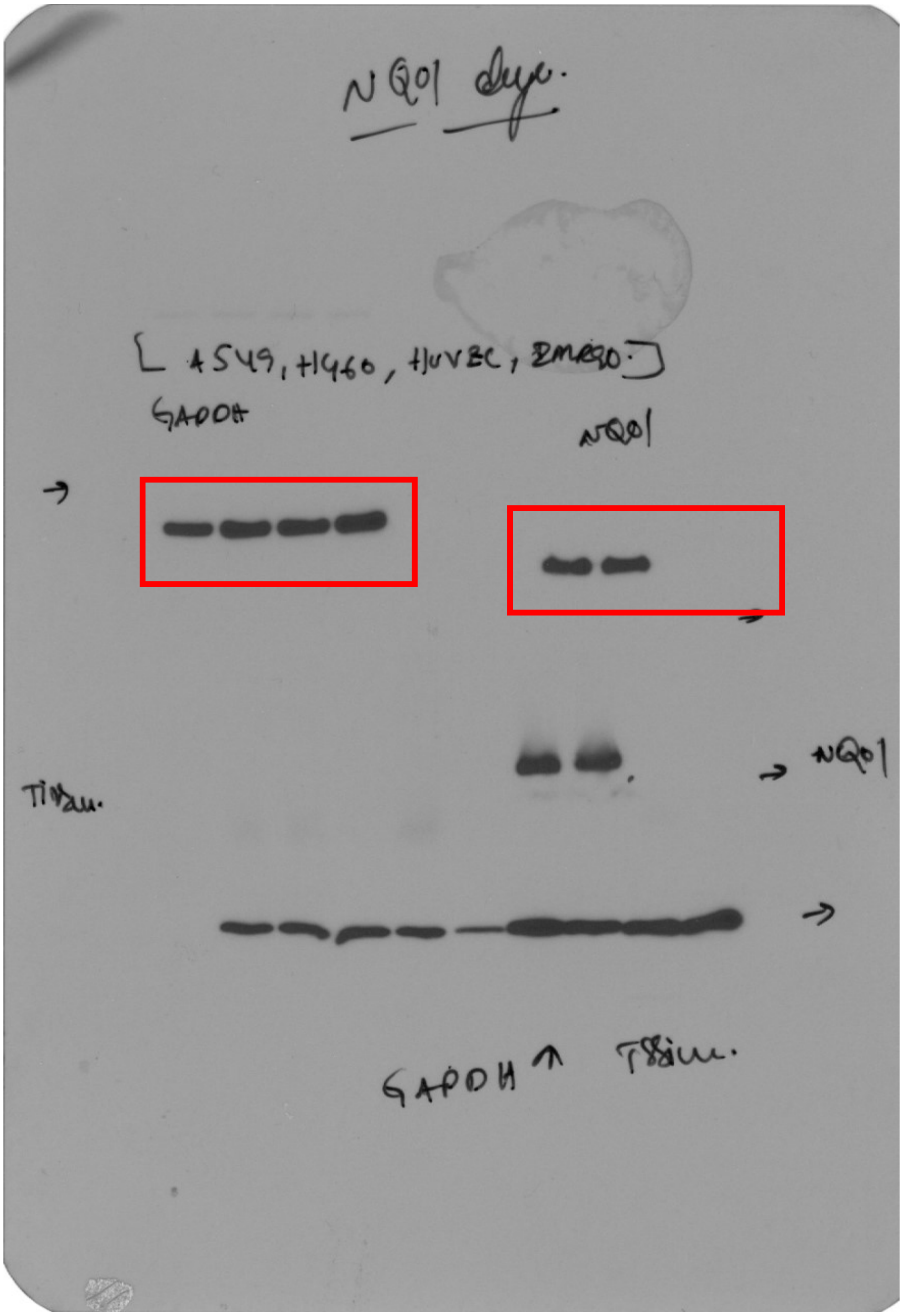

Figure S9. Corresponding to Figure 3B present in manuscript Expression of NQO1 in different cells

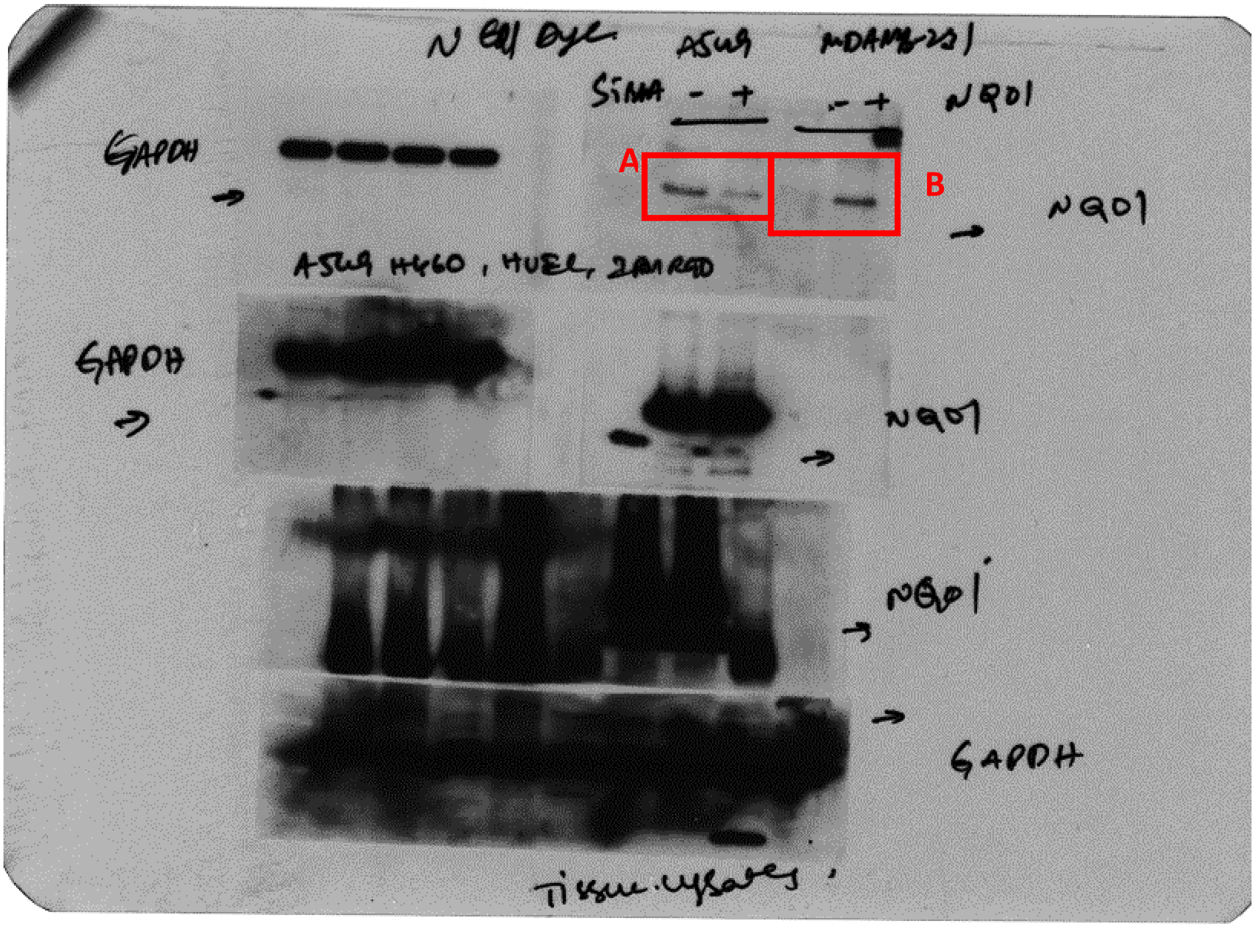

Figure S10. Corresponding to Figure 4C (A; SiRNA knockdown of NQO1 A549 cells) and Figure 4E (B; Expression of NQO1 in MDA-MB-231) present in manuscript

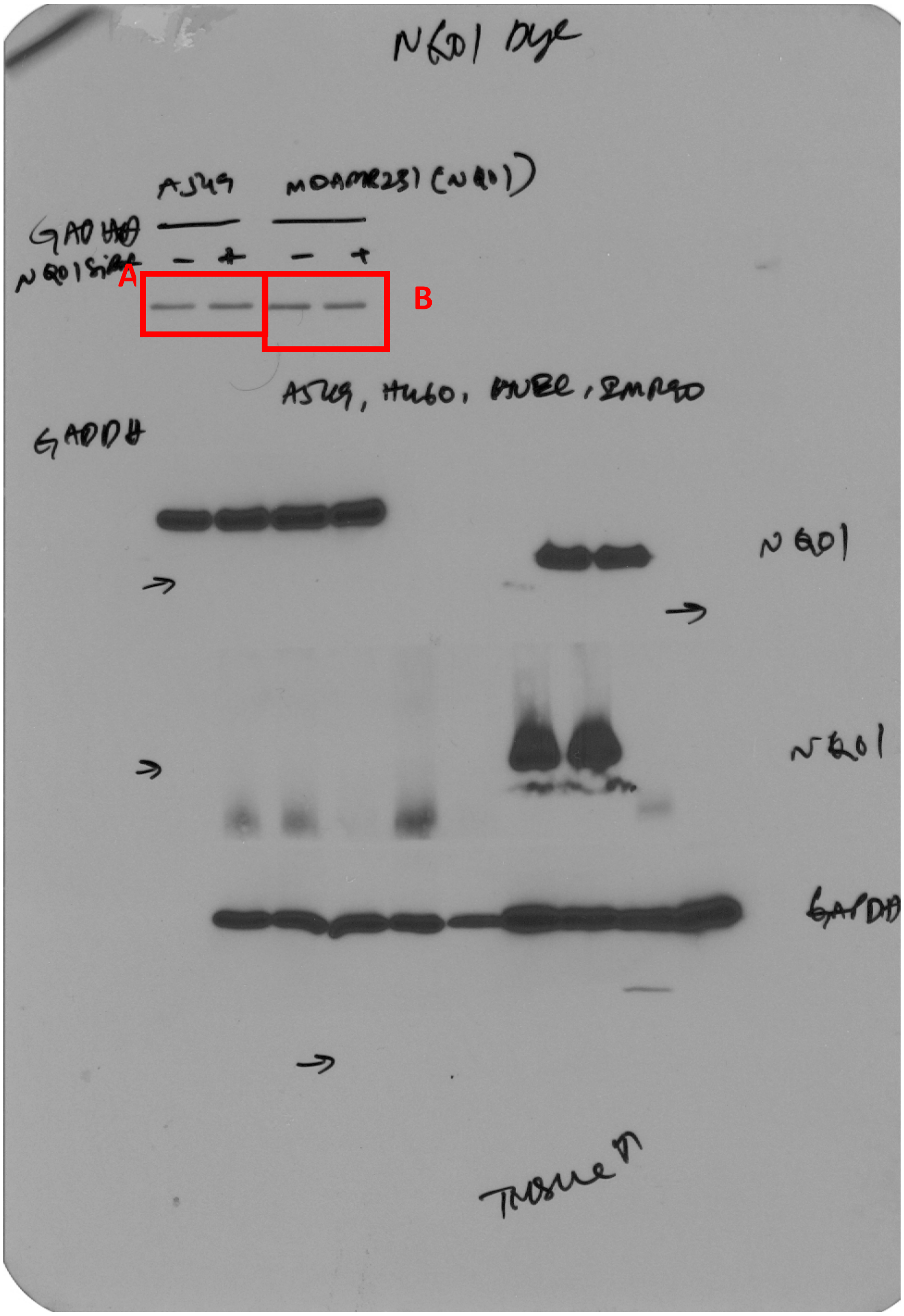

Figure S11. Loading controls Corresponding to Figure 4C (A; SiRNA knockdown of NQO1 A549 cells) and Figure 4E (B; Expression of NQO1 in MDA-MB-231) present in manuscript

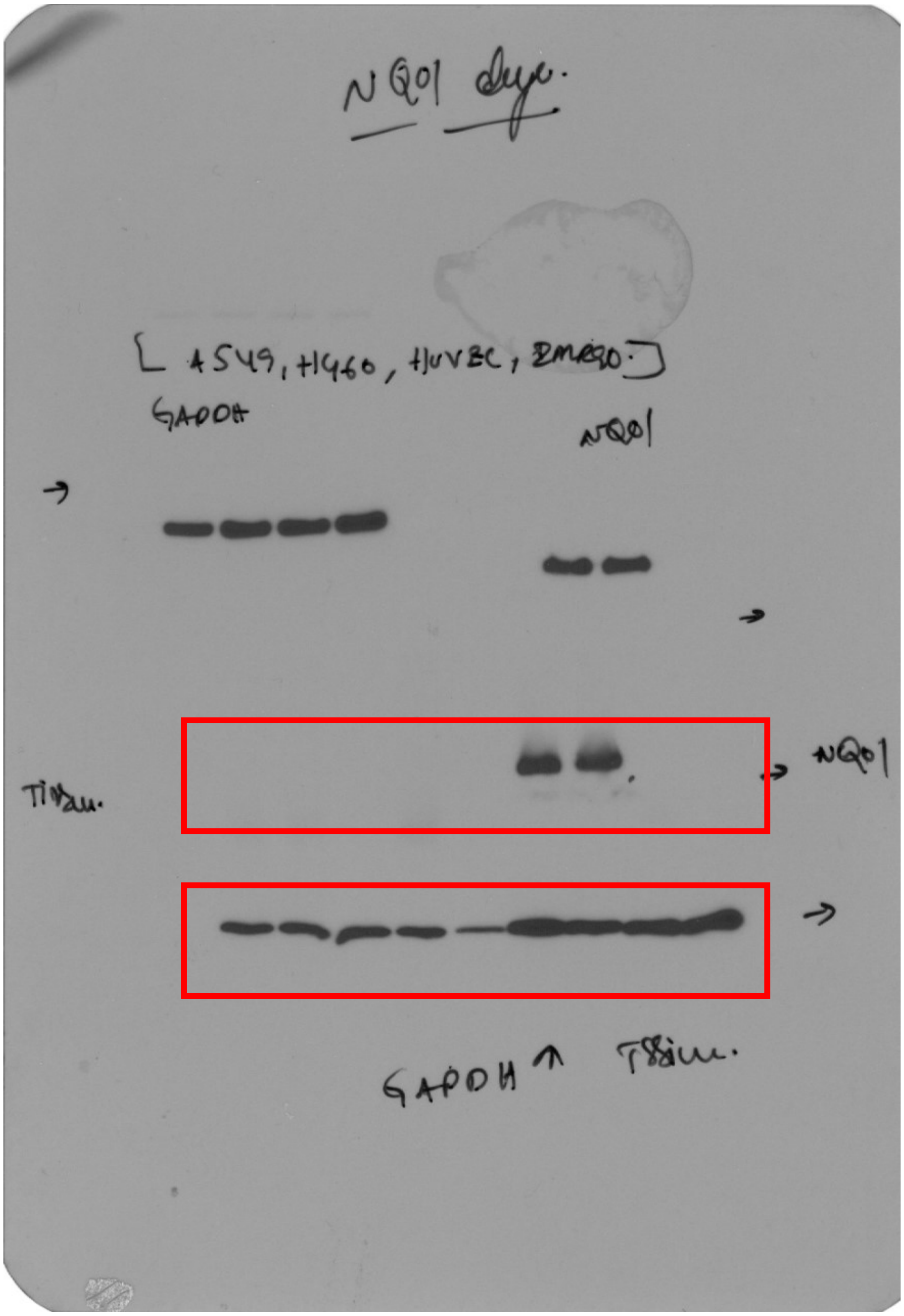

Figure S12. Corresponding to Figure 5C present in manuscript; expression of NQO1 in different tissue lysates

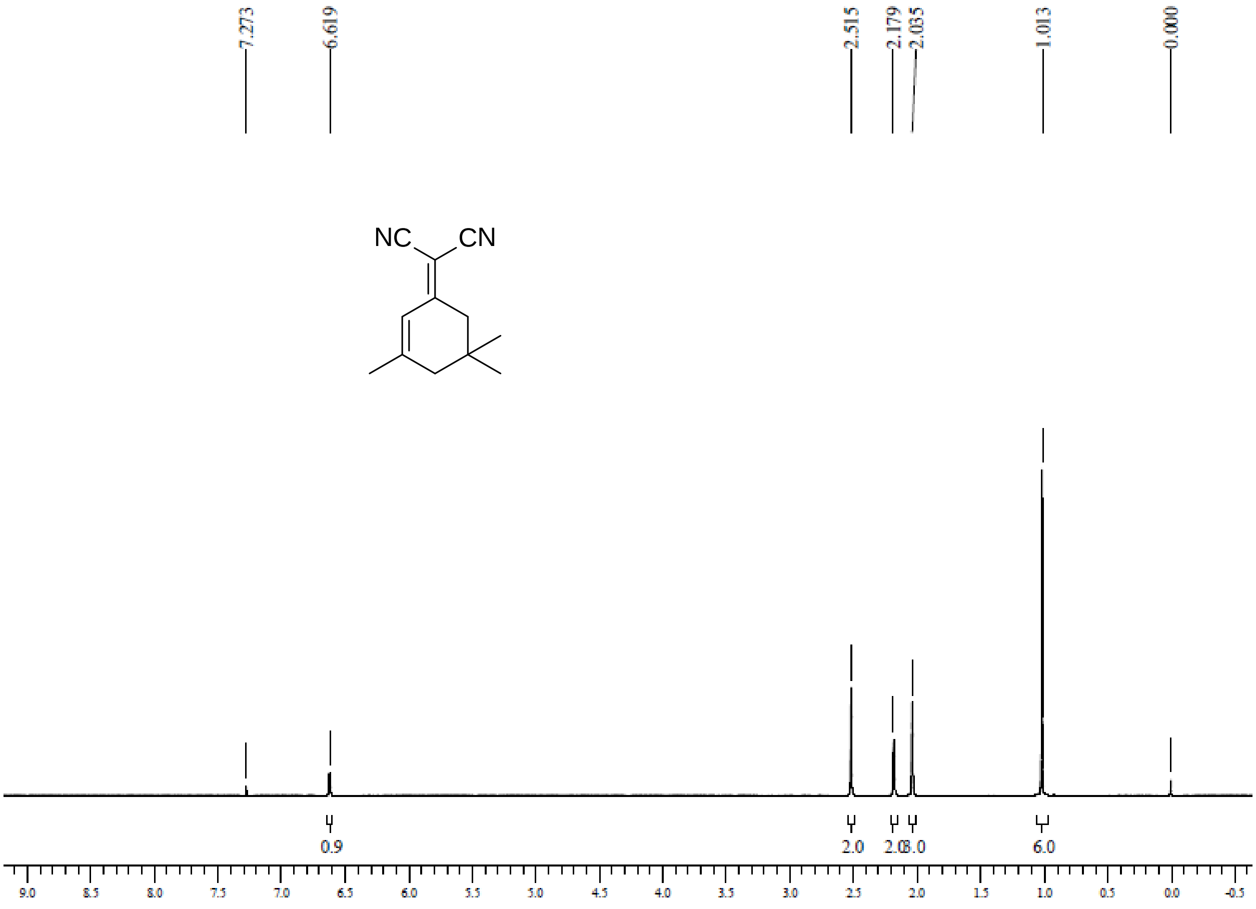

Figure S13. ^{1}HNMR Spectrum of 2-(3,5,5-trimethylcyclohex-2-en-1-ylidene)malononitrile (2)

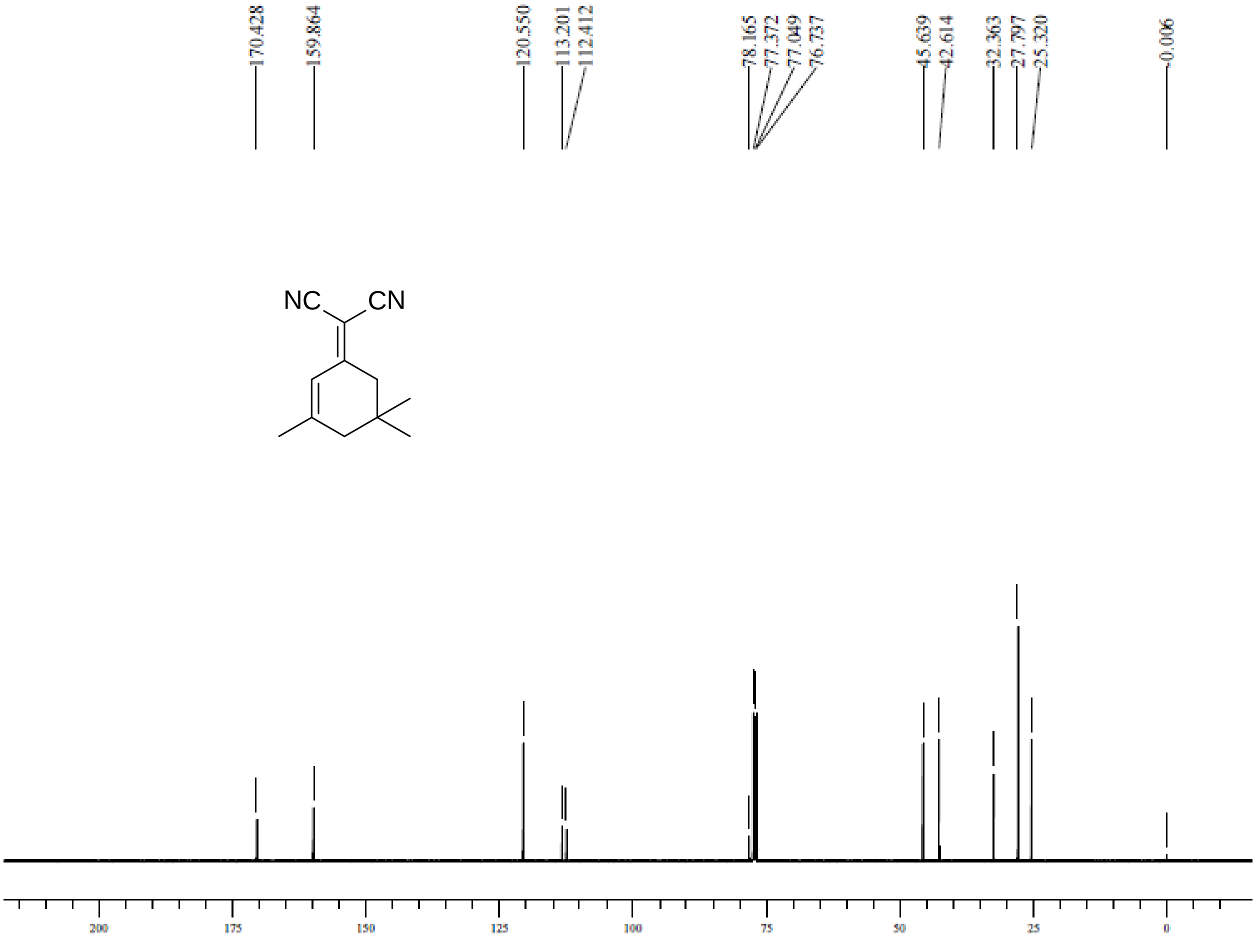

Figure S14. ^{13}CNMR Spectrum of 2-(3,5,5-trimethylcyclohex-2-en-1-ylidene)malononitrile (2)

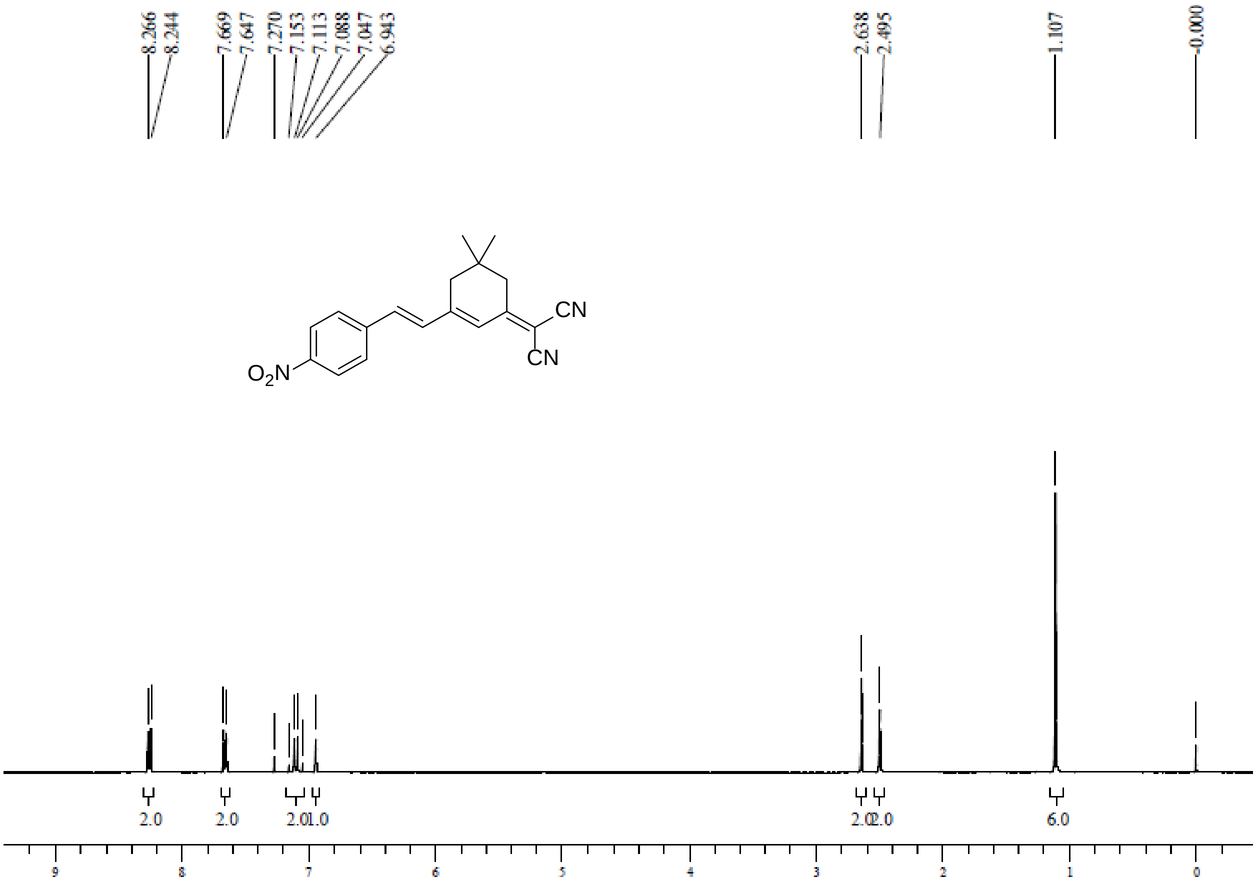

Figure S15.^{1}HNMR Spectrum of (E)-2-(5,5-dimethyl-3-(4-nitrostyryl)cyclohex-2-en-1-ylidene)malononitrile (3)

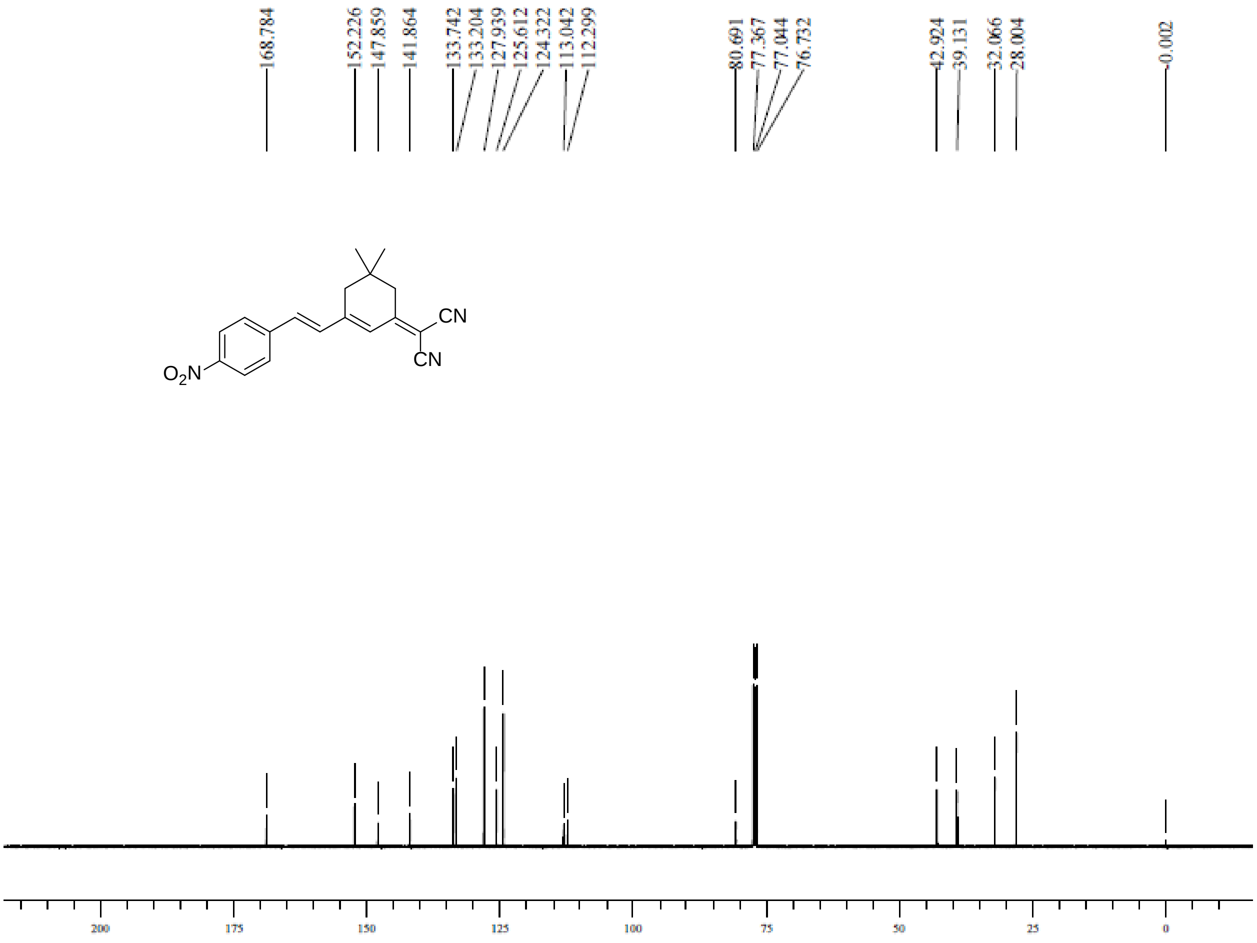

Figure S16.^{13}CNMR Spectrum of (E)-2-(5,5-dimethyl-3-(4-nitrostyryl) cyclohex-2-en-1-ylidene) malononitrile (3)

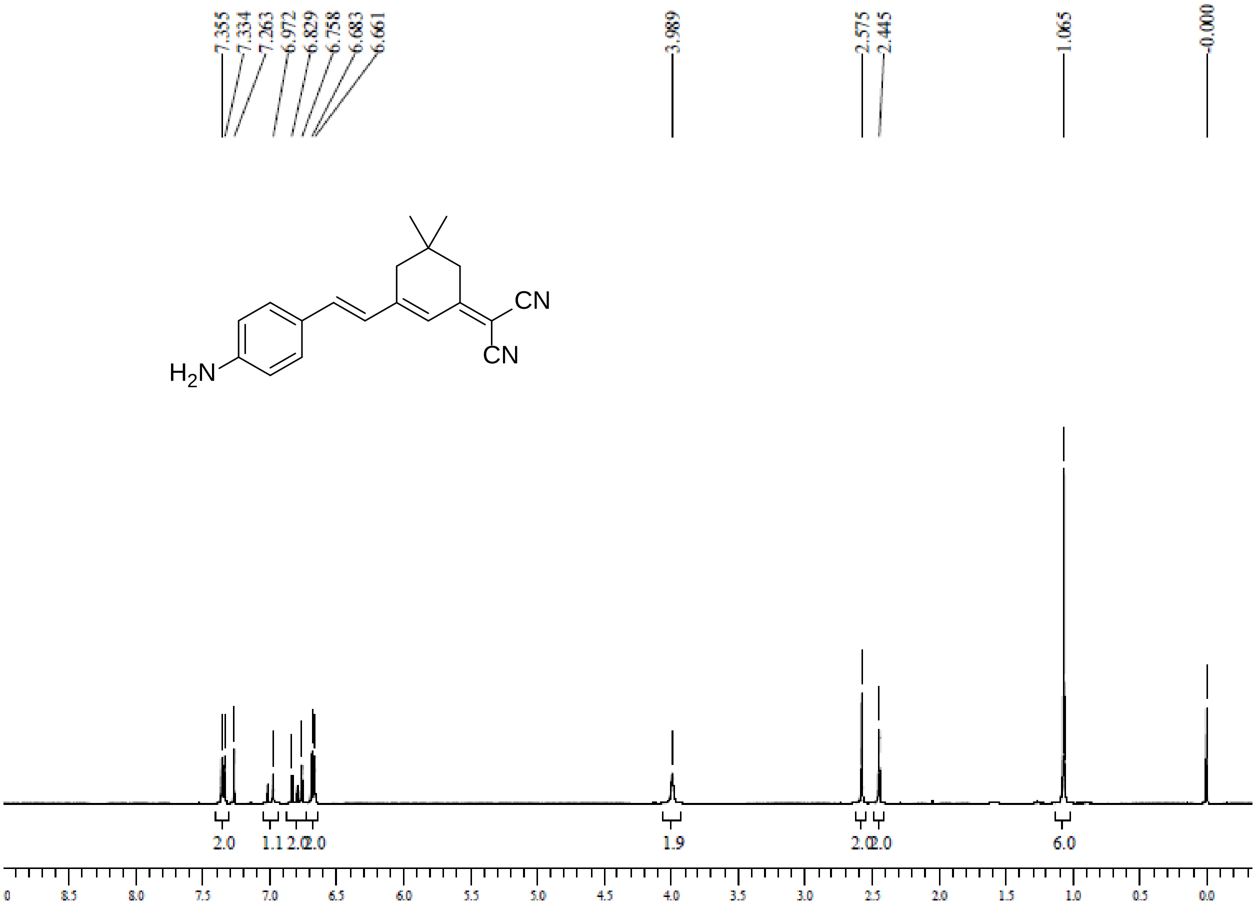

Figure S17.^{1}HNMR Spectrum of (E)-2-(3-(4-aminostyryl)-5,5-dimethylcyclohex-2-en-1-ylidene) malononitrile (4, DCl)

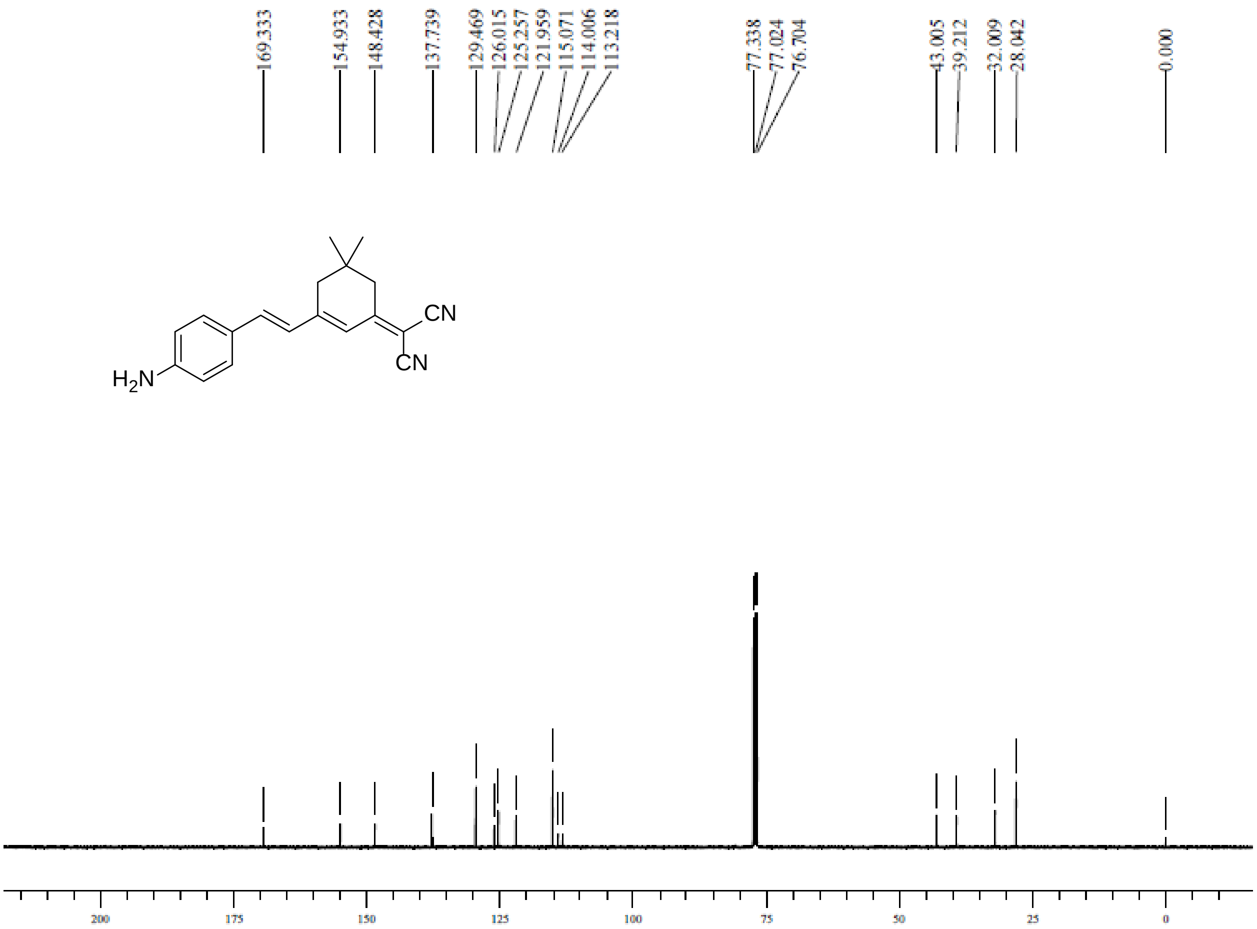

Figure 18. ^{13}CNMR Spectrum of (E)-2-(3-(4-aminostyryl)-5,5-dimethylcyclohex-2-en-1-ylidene) malononitrile (4, DCl)

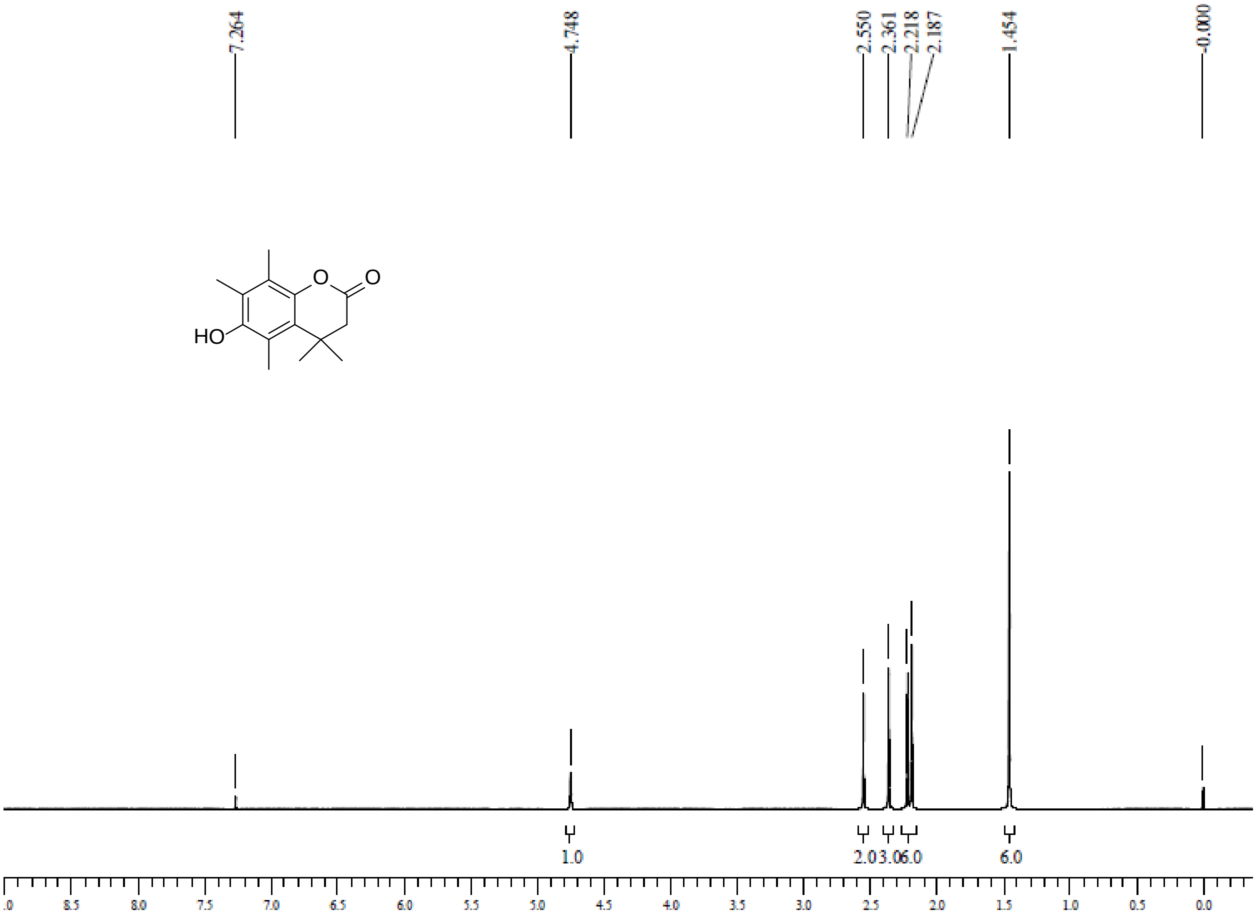

Figure S19. ^{1}HNMR Spectrum of 6-hydroxy-4,4,5,7,8-pentamethylchroman-2-one (6)

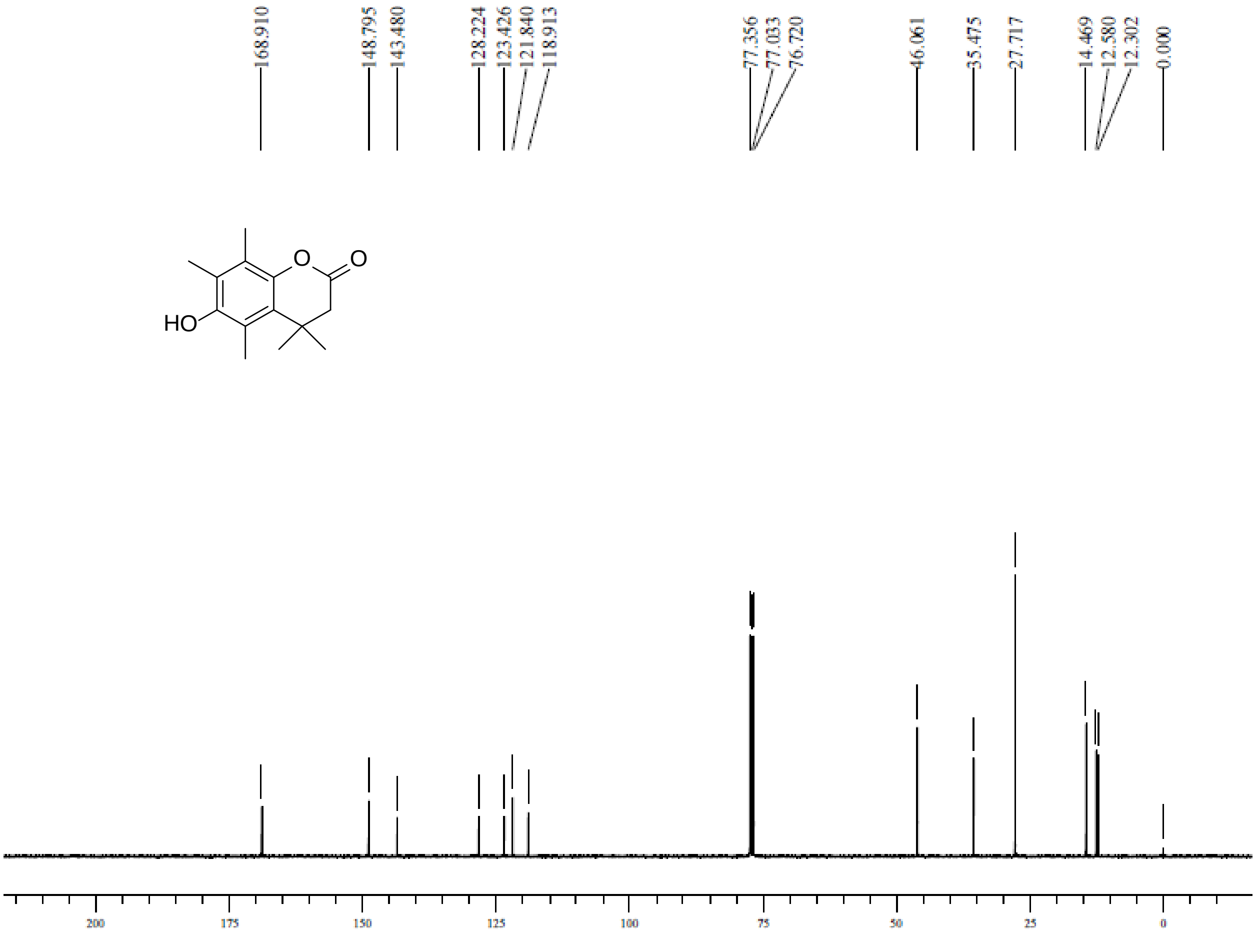

Figure S20. ^{13}CNMR Spectrum of 6-hydroxy-4,4,5,7,8-pentamethylchroman-2-one (6)

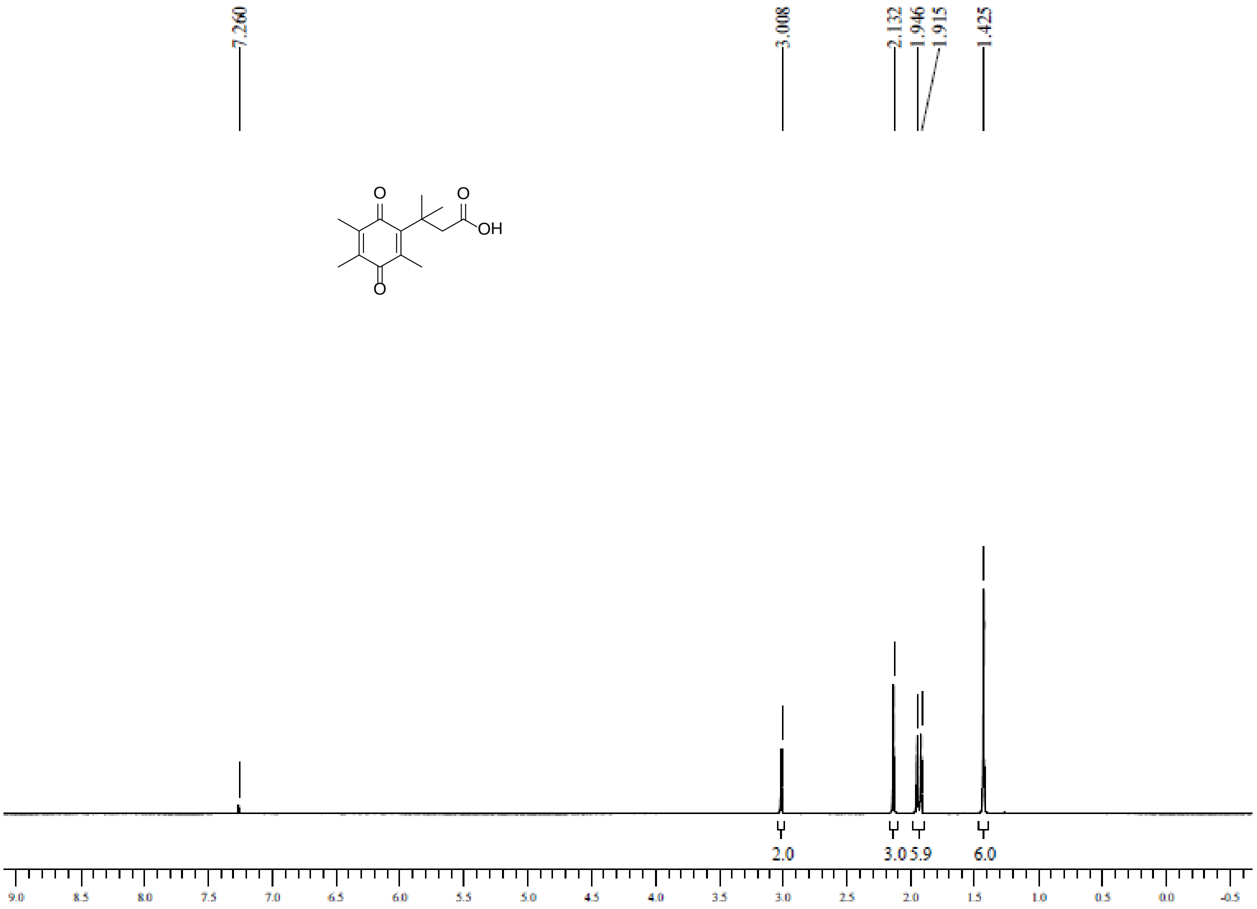

Figure S21. ^{1}HNMR Spectrum of 3-methyl-3-(2,4,5-trimethyl-3,6-dioxocyclohexa-1,4-dien-1-yl)butanoic acid (7, QPA)

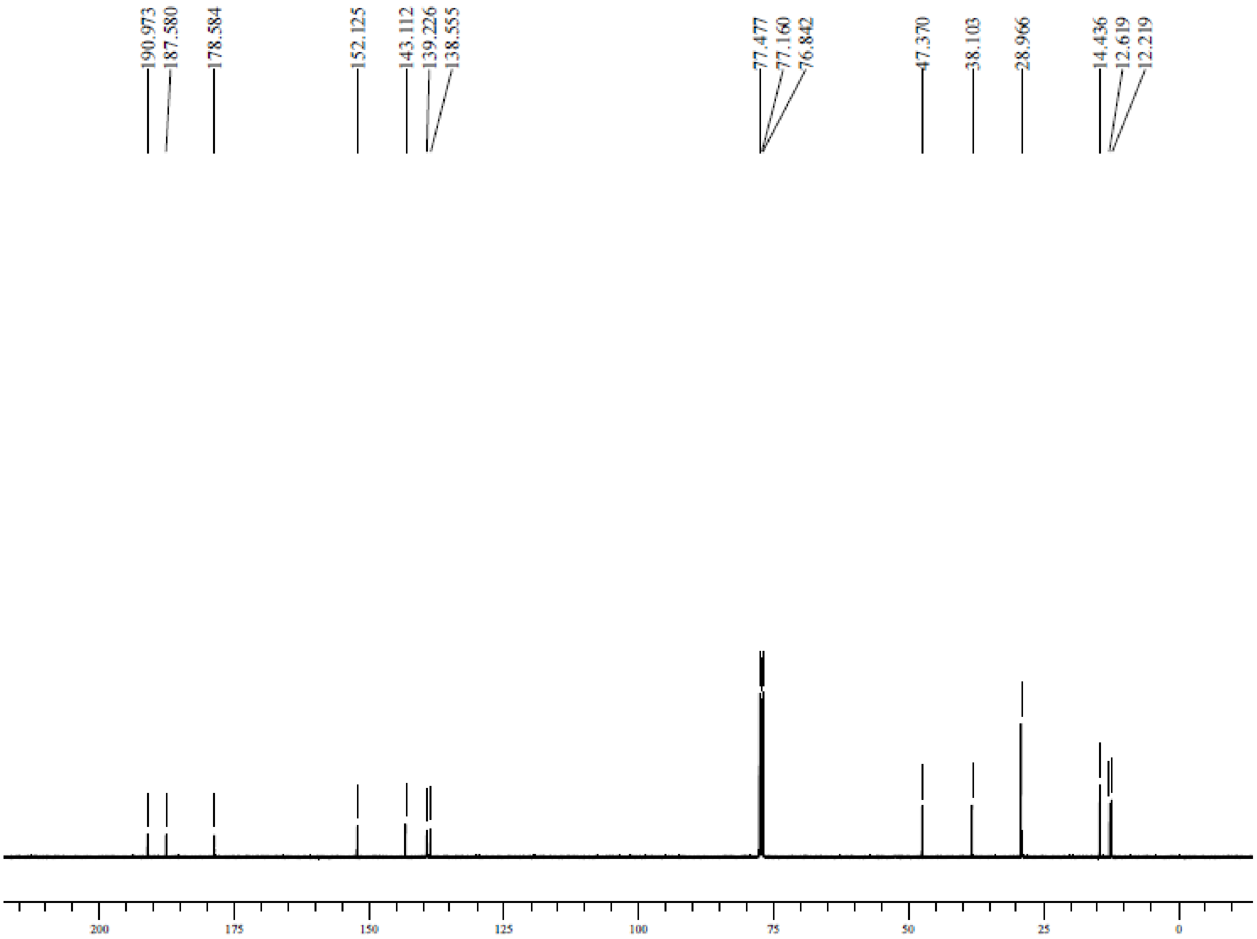

Figure S22. ^{13}CNMR Spectrum of 3-methyl-3-(2,4,5-trimethyl-3,6-dioxocyclohexa-1,4-dien-1-yl)butanoic acid (7, QPA)

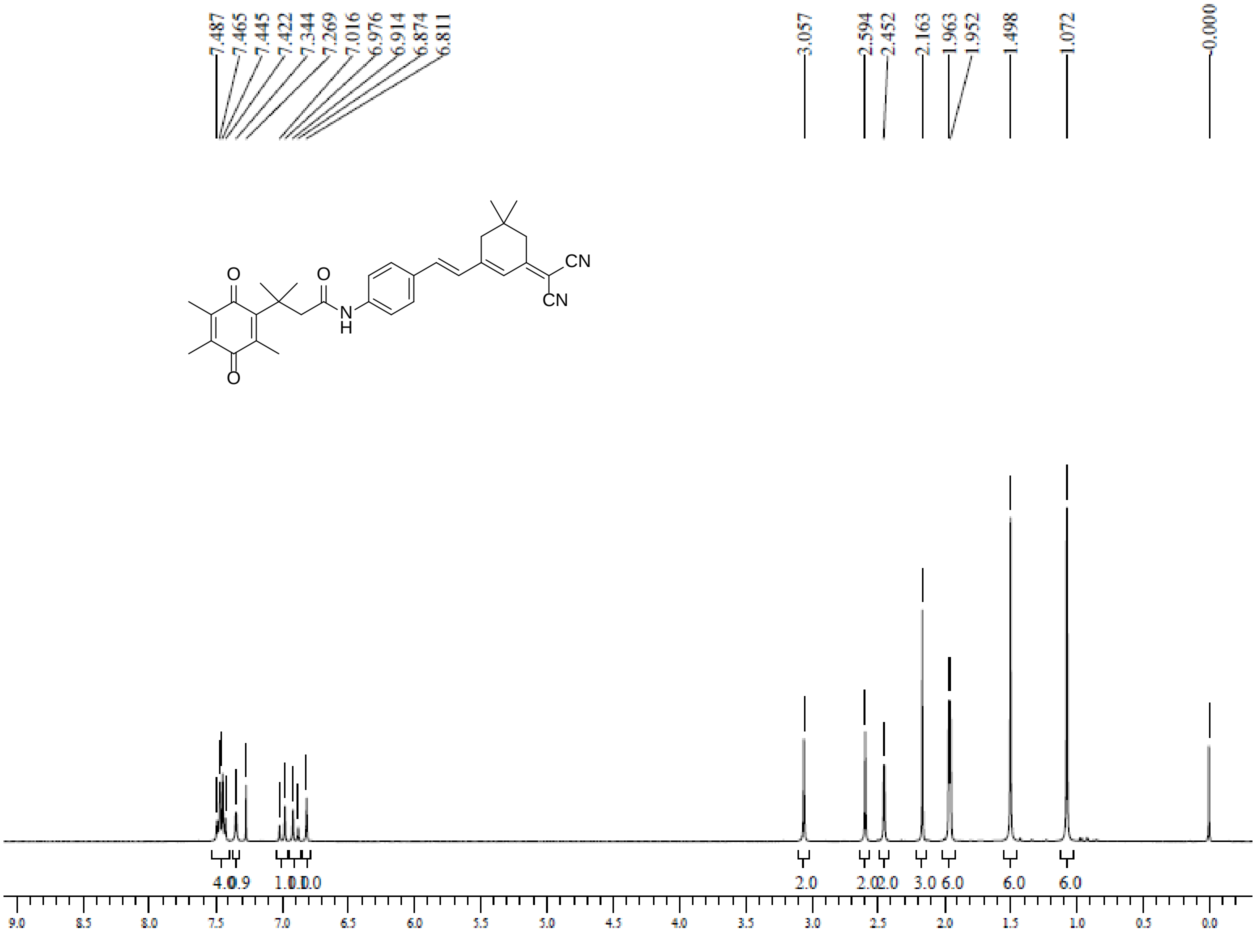

Figure S24. ^{1}HNMR Spectrum of (E)-N-(4-(2-(3-(dicyanomethylene)-5,5-dimethylcyclohex-1-en-1-yl) vinyl) phenyl)-3-methyl-3-(2,4,5-trimethyl-3,6-dioxocyclohexa-1,4-dien-1-yl) butanamide (NQ-DCI)

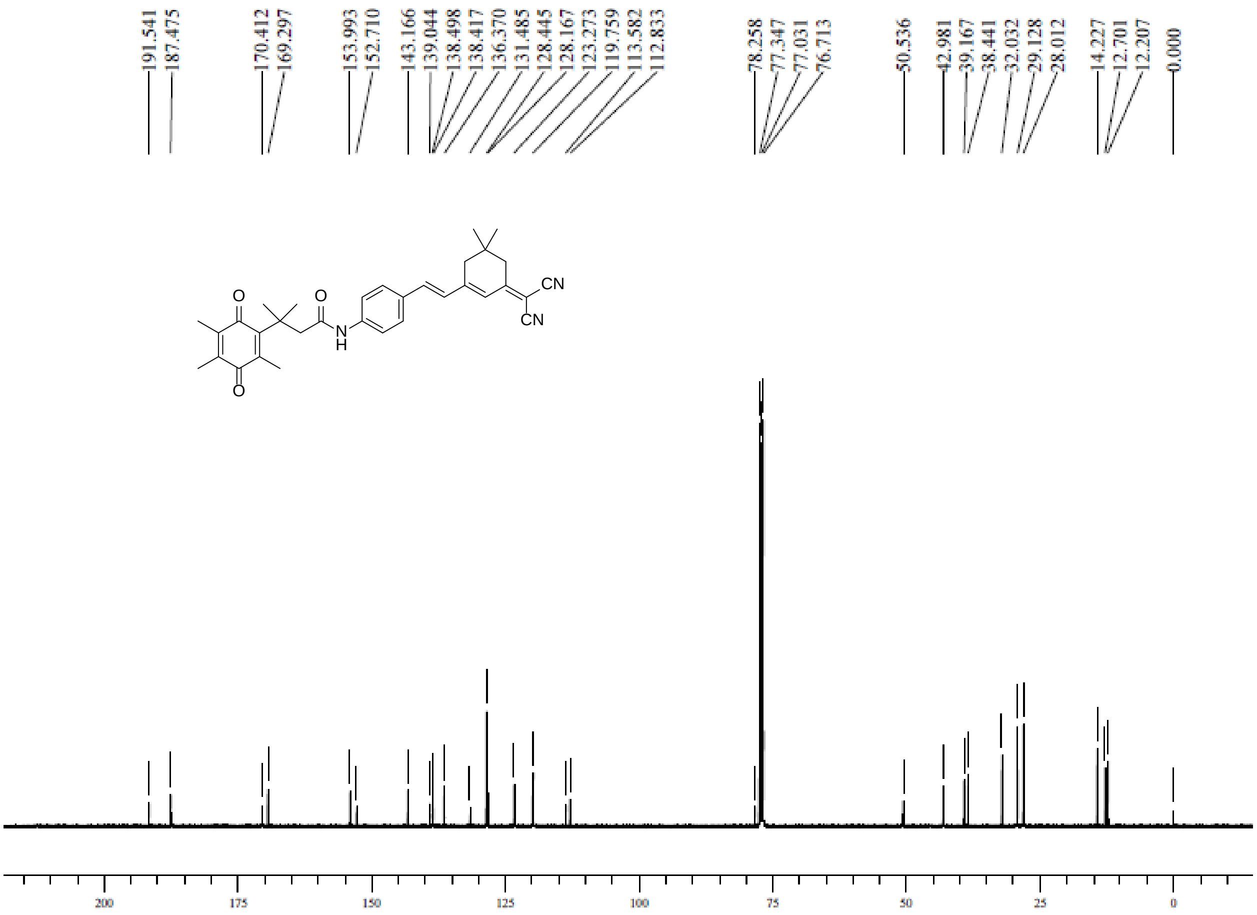

Figure S25. ^{13}CNMR Spectrum of (E)-N-(4-(2-(3-(dicyanomethylene)-5,5-dimethylcyclohex-1-en-1-yl) vinyl) phenyl)-3-methyl-3-(2,4,5-trimethyl-3,6-dioxocyclohexa-1,4-dien-1-yl) butanamide (NQ-DCI).
